# Multiple Argonaute complexes orchestrate epigenetic silencing in the Arabidopsis germline

**DOI:** 10.1038/s41467-026-77326-1

**Published:** 2026-09-12

**Authors:** Jincheng Long, Chao Yang, Shujuan Xu, Joke De Jaeger-Braet, Wenjing She, Seijiro Ono, James Walker, Ygor De Souza-Vieira, Arp Schnittger, Xiaoqi Feng

**Affiliations:** 1 Department of Cell and Developmental Biology, John Innes Centre, Norwich, UK; 2 State Key Laboratory of Plant Trait Design, CAS Center for Excellence in Molecular Plant Sciences, Shanghai Institute of Plant Physiology and Ecology, Chinese Academy of Sciences, Shanghai, China; 3 Institute of Science and Technology Austria, Klosterneuburg, Austria; 4 Department of Developmental Biology, Institute of Plant Science and Microbiology, University of Hamburg, Hamburg, Germany; 5 Present Address: The Salk Institute for Biological Studies, La Jolla, CA USA

**Keywords:** Plant reproduction, Plant cell biology, DNA methylation, Gene silencing

## Abstract

Small RNAs (sRNAs) are widespread across diverse flowering plant species and play important roles regulating reproduction and environmental responses. However, how sRNAs exert their function is largely unknown, likely due to their typical origin in non-genic regions, the lack of distinct gene targets, and the sparse characterization of their associated Argonaute (AGO) effector proteins. Here we show that *Arabidopsis* male reproductive sRNAs associate with AGO4, 5, 6 and 9, all of which are enriched in meiocytes along with RNA Polymerase V (Pol V). This enables these sRNAs to methylate loci with imperfect sequence homology. AGO4, AGO6 and AGO9 predominantly load 24-nucleotide (nt) Pol IV-derived sRNAs and mediate DNA methylation in meiocytes in a dosage-dependent manner. Despite closer homology to microRNA-associated AGO1 and AGO10, AGO5 primarily binds CLSY3- and Pol IV-derived 21-24 nt sRNAs. Transcriptomic and methylomic analyses of meiocytes from single and compound *ago* mutants demonstrate distinct yet complementary roles of AGO4/6/9 and AGO5 in regulating protein-coding genes and silencing transposons in the germline. These molecular disruptions are accompanied by significant meiotic defects. Together, our findings reveal how AGO4/6/9-mediated DNA methylation and AGO5, likely acting through post-transcriptional gene silencing, work together to promote male germline development.

## Abstract (web summary)

Long *et al*. reveal how reproductive small RNAs engage multiple Argonaute proteins to coordinate DNA methylation and post-transcriptional gene silencing in the Arabidopsis germline, supporting meiosis and fertility.

## Introduction

Small RNAs (sRNAs) are essential regulators of gene expression, genome stability and immunity in eukaryotes, contributing to diverse biological processes from development to stress response^1^. In plants, sRNAs typically range from 20 to 24 nucleotides (nt) in length and are primarily categorized into microRNAs (miRNAs) and small interfering RNAs (siRNAs)^2^. Among these, 24-nucleotide (nt) siRNAs are the most abundant in plant genomes and play a central role in RNA-directed DNA methylation (RdDM)^3^.

RdDM is composed of two interconnected branches that form a positive feedback loop: the siRNA biogenesis branch, also known as the RNA Polymerase IV (Pol IV) pathway, and the DNA methylation branch, also known as the Pol V pathway^3^. The siRNA biogenesis is initiated when Pol IV is recruited to a genomic locus through the action of four CLASSY (CLSY) proteins^4–6^. CLSY1-4 exhibit tissue-specificity and govern the recruitment of Pol IV to different genomic loci^5,7^. Pol IV transcribes a short non-coding RNA and forms a complex with RNA-DEPENDENT RNA POLYMERASE 2 (RDR2), which synthesizes the complementary strand, forming double-stranded RNA (dsRNA)^8–10^. dsRNA precursors are then released from Pol IV-RDR2 complex and processed by DICER-LIKE 3 (DCL3) into 24-nt siRNA duplexes^11,12^. In the Pol V pathway, one strand of the siRNA duplex is loaded onto an ARGONAUTE (AGO) protein, typically AGO4/6/9^13,14^. This siRNA-AGO complex then binds to a complementary transcript generated by Pol V, enabling the recruitment of DOMAINS REARRANGED METHYTRANSFERASE2 (DRM2) to methylate the genomic region^3^. DRM2 catalyzes cytosine methylation across all sequence contexts (CG/CHG/CHH, where H = A, T, or C)^15^. RdDM is self-reinforcing, as siRNAs guide DNA methylation through the Pol V pathway, while DNA methylation, in turn, recruits the Pol IV pathway to generate siRNAs^3^.

Besides AGO4, 6 and 9, the *Arabidopsis* genome encodes six other AGO proteins (excluding the pseudogene AGO8), which are classified into two additional clades based on their sequence similarity: AGO1/5/10 and AGO2/3/7^16^. The AGO1/5/10 clade proteins, particularly AGO1, which plays a central role in development^17,18^, are primarily associated with miRNAs, which mediate post-transcriptional gene silencing (PTGS) through transcript cleavage or translational repression^2^. Among the AGO2/3/7 clade proteins, AGO2 is a key component of antiviral defense, and *ago2* mutants exhibit increased susceptibility to RNA virus infections^19^. AGO3, which shares approximately 70% sequence identity with AGO2, has been implicated in the RdDM pathway^20,21^. In *Arabidopsis*, AGO7 plays a crucial role in developmental regulation, particularly through the production of transacting small interfering RNAs (ta-siRNAs) via its interaction with miR390^22^. TasiRNAs participate in PTGS by guiding the cleavage of target mRNAs, thereby playing crucial roles in immune defense and developmental regulation^2,23–25^.

Although most AGOs have been well studied, the sRNAs associated with AGO5 and their biological functions are unclear. AGO5 has been reported to exhibit specific expression patterns in reproductive cells, including the egg and sperm, as well as in subepidermal cells of the shoot apical meristem (SAM), precursor cells giving rise to lateral (incl. sex) organs^26,27^. Interestingly, despite its close relationship to AGO1 and 10, a recent study indicates that AGO5 associates with sRNAs derived from transposable elements (TEs) in the SAM, suggesting a role in RdDM^26^. However, the features and biological functions of AGO5-associated sRNAs are largely unexplored, and the significance of AGO5’s specific accumulation in germ cells and related lineages remains unclear.

Specialized siRNAs accumulate at high levels during male reproductive development across diverse flowering plant species. In monocots such as maize and rice, reproductive-phase secondary siRNAs (phasiRNAs) are generated from intergenic *PHAS* loci^2,28^. These phasiRNAs are transcribed by RNA polymerase II (Pol II), cleaved by miRNAs, and subsequently converted into double-stranded RNA (dsRNA) by RDR6^2,28^. The resulting dsRNA precursors are processed by DCL enzymes into phasiRNAs with a characteristic phased pattern. There are two major groups of reproductive phasiRNAs: 21-nt phasiRNAs, generated by DCL4 following miR2118 cleavage, and 24-nt phasiRNAs, produced mainly by miR2275 and DCL5^29–32^. These two groups exhibit distinct spatiotemporal expression patterns during male reproductive development. While 21-nt phasiRNAs accumulate pre-meiotically^32,33^, most 24-nt phasiRNAs are expressed during meiosis and persist through post-meiotic development^32^. In maize, 21-nt phasiRNAs are absent in mutants lacking epidermal tissue, indicating that these siRNAs are synthesized in these cells^32^. In contrast, the tapetum — but not the male meiocytes — is required for 24-nt phasiRNA production^32^. DCL5-specific expression in the tapetum, coupled with the highest accumulation of 24-nt phasiRNAs in meiocytes, indicates that these tapetum-derived 24-nt phasiRNAs are transported intercellularly into germ cells^34^.

21- and 24-nt phasiRNAs are important for male fertility, especially under environmental stress^31,35–38^, highlighting their functional significance in reproductive development. Despite their importance, the molecular functions of phasiRNAs remain poorly understood due to several challenges. First, the absence of clear genic targets in the genome complicates functional characterization. Second, our knowledge of the AGO proteins that associate with phasiRNAs is limited. Reproductive 21-nt phasiRNAs in rice have been shown to load into MEL1 (the rice AGO5 homolog) and OsAGO1d, which regulate meiotic progression and cold stress response, respectively^35,36,38–41^. However, the identity of AGO proteins that directly load meiotic 24-nt phasiRNAs and mediate their biological functions remain unclear. Third, progress is further hampered by the complexity of monocot genomes and the time-consuming nature of genetic studies in these species.

Although 24-nt reproductive phasiRNAs have also been reported from eudicot species^42^, *Arabidopsis* is an exception, as it lacks both reproductive 21-nt and 24-nt phasiRNAs^28^. However, we previously discovered that *Arabidopsis* anthers accumulate a high abundance of Pol IV-produced 24-nt siRNAs during early meiosis^43^. These siRNAs originate primarily from intergenic and repetitive regions that are hypermethylated in all sequence contexts (CG, CHG and CHH) in the tapetum; as 70% of these source loci overlap TEs, these are named HyperTEs (797 loci in total)^43^. Genetic mosaic analysis demonstrated that these tapetal siRNAs move into meiocytes, where they induce methylation at HyperTEs via perfect sequence complementarity, as well as at homologous loci with up to three mismatches^43^. Many of these imperfectly matched target loci overlap genes and are hypermethylated in the meiocyte; hence, we named them MetGenes (469 loci in total)^43^. MetGene methylation regulates gene expression and splicing in meiocytes, thereby promoting meiosis^44^. The specific ability of the tapetum to generate these siRNAs is attributed to the tapetum-specific expression of CLSY3, which recruits Pol IV to HyperTEs^43^. Although the tapetum is similarly enriched in HyperTE-derived siRNAs, they fail to induce methylation at MetGenes^43^. This indicates that unknown molecular features specific to meiocytes are required for the broad targeting of loci with sequence mismatches. Understanding this meiocyte-specific targeting mechanism is crucial for elucidating germline epigenetic regulation and may also shed light on the biological functions of phasiRNAs. Given the spatiotemporal similarity between HyperTE-derived siRNAs and 24-nt phasiRNAs, it is conceivable that they play analogous regulatory roles in plant reproductive development.

In this study, we aimed to elucidate the special molecular mechanism in meiocytes responsible for DNA methylation reprogramming by identifying the AGO effector proteins involved. We comprehensively examined the spatial distribution of 9 AGO proteins in *Arabidopsis* meiotic anthers and found that AGO4, AGO5, AGO6, and AGO9 are abundantly expressed in meiocytes. RNA immunoprecipitation coupled with sequencing (RIP-seq) revealed that all of these AGOs predominantly bind siRNAs derived from Pol IV-dependent siRNA clusters. Using genetic analyzes, we further demonstrated that the biogenesis of AGO5-loaded siRNAs is dependent on Pol IV and CLSY3. We discovered that the Pol V pathway of RdDM is elaborated in the meiocyte, including an elevated expression of AGO4/6/9 and Pol V itself, and further showed that this elaboration is required for targeting MetGenes with sequence mismatches. In comparison, AGO5 plays a relatively minor, if any, role in meiocyte RdDM. Transcriptome and DNA methylome analyzes of meiocytes from various *ago* single and compound mutants further demonstrate that AGO5—likely through PTGS—and AGO4/6/9-mediated DNA methylation play important roles in regulating the transcription of protein-coding genes and silencing TEs in the male germline. Meiotic phenotyping revealed significant meiotic defects in *ago5* and *ago4,6,9* mutants. Taken together, our results show that *Arabidopsis* germline siRNAs are incorporated into multiple AGO proteins, which play distinct yet complementary regulatory roles in male meiocytes.

## Results

### AGO4, 5, 6 and 9 are abundantly expressed in *Arabidopsis* meiocytes

**Fig. 1:**
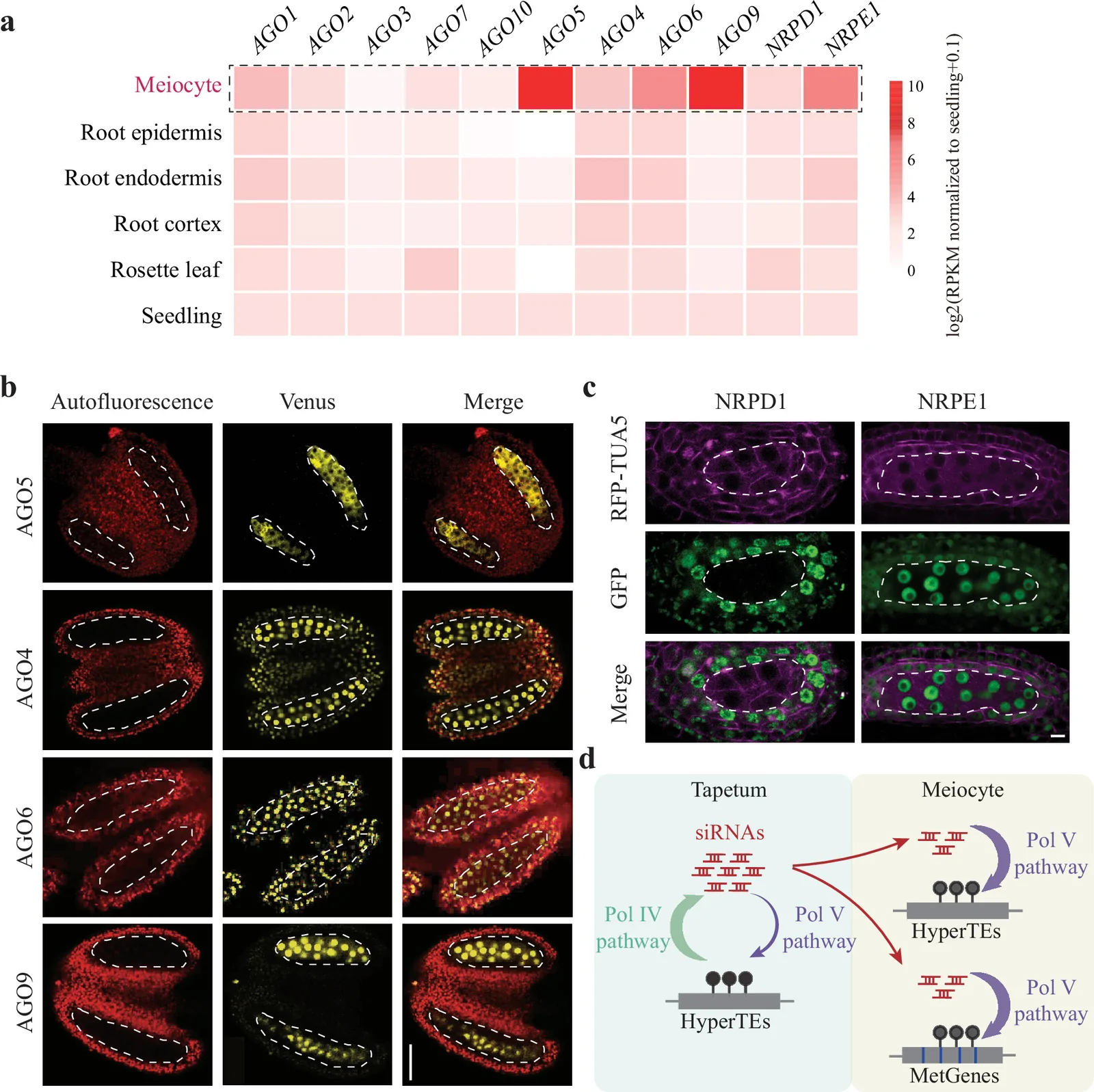
AGO4, AGO5, AGO6, and AGO9 are abundantly enriched in meiocyte. **a** Heatmap illustrating transcription levels of indicated genes across various tissues and cell types in *Arabidopsis*. **b** Confocal images depicting localization patterns of Venus-tagged AGO4, AGO5, AGO6, and AGO9 in Stage 6 anthers. Scale bar, 25 μm. **c** Confocal images showing localization patterns of NRPD1-GFP and NRPE1-GFP in Stage 6 anthers. Both lines are in the *pRPS5A::RFP-TUA5* background, which labels the microtubule cytoskeleton. Scale bar, 5 μm. **d** Cartoon depicting the decoupling of Pol IV and Pol V pathways in the meiocyte. In the tapetum, the Pol IV pathway (e.g., Pol IV, CLSY3) is highly enriched, producing large quantities of siRNAs that are transported into neighboring meiocytes. In the meiocyte, the Pol IV pathway is inactive, while the Pol V pathway is highly enriched (e.g., Pol V, AGO4, AGO6 and AGO9), allowing tapetal siRNAs to target both HyperTEs and MetGenes. The absence of Pol IV pathway in meiocytes prevents the production of perfectly matching siRNAs from MetGenes, limiting the spread of methylation to additional targets. Blue lines on the MetGene model indicate nucleotide mismatches.

To identify AGO proteins highly expressed in meiocytes, we analyzed publicly available RNA-sequencing (RNA-seq) data^44–47^ for AGO family members *(AGO1-AGO10)* in meiocytes and somatic tissues. *AGO5*, *AGO6*, and *AGO9* exhibited higher expression levels in meiocytes compared to somatic tissues (Fig. 1a). However, as transcriptional levels do not always reflect protein abundance, we generated AGO1-AGO10 reporter lines (excluding AGO8, a pseudogene), in which the AGO open reading frames were fused with a Venus tag at the N-terminus and driven by their native promoters (Supplementary Fig. 1a). Confocal imaging of stage 6 anthers (meiosis stage; anther stages defined in ref.^48^) from these reporter lines revealed that AGO3, AGO7 and AGO10 were undetectable in the meiocyte, while AGO1 and AGO2 exhibited weak cytoplasmic signals relative to surrounding anther wall layers (Supplementary Fig. 1b). In contrast, AGO4, AGO5, AGO6, and AGO9 were strongly expressed in the meiocytes, with AGO4, AGO6 and AGO9 localized to the nucleus while AGO5 to the cytoplasm (Fig. 1b). Notably, AGO4 and AGO6 were also expressed in the tapetum and other somatic cell layers, though at lower levels than in the meiocyte (Fig. 1b). In contrast, AGO5 and AGO9 were strictly confined to meiocytes, consistent with a previous report^26^ (Fig. 1b). Therefore, while canonical RdDM in somatic tissues predominantly relies on AGO4^49^, RdDM in meiocytes seems to involve a broader range of AGO proteins, including the distinct and robust expression of AGO9, and elevated levels of AGO4 and AGO6.

The highly elevated levels of AGO4/6/9 in meiocytes suggest that the increased dosage of Pol V pathway components could contribute to the pronounced off-target methylation effects. To test this idea, we constructed Pol V-GFP reporter lines using Pol V’s largest subunit, NRPE1, driven by its own promoter (*pNRPE1::NRPE1-GFP*) and examined its localization pattern in meiotic-stage anthers. Consistent with our hypothesis, we found that the NRPE1-GFP signal strongly localizes in the nucleus of meiocyte, at a much higher level than that in the tapetum and somatic tissues (Fig. 1c). In parallel, we also created reporter lines of Pol IV using its largest subunit NRPD1 (*pNRPD1::NRPD1-GFP*) and observed NRPD1-GFP was undetectable in meiocytes, but prominent in the nucleus of parietal cell layers, especially the tapetum (Fig. 1c). This result is consistent with our previous finding that meiocytes do not produce their own 24-nt siRNAs, and that 24-nt siRNAs in meiocyte are transported from the tapetum^43^. Typically, RdDM operates as a self-reinforcing loop between the Pol IV and Pol V pathways^3^. Our findings demonstrate that these pathways are spatially decoupled in the anther, with elevated Pol IV activity in the tapetum generating abundant siRNAs while AGO and Pol V activities are strongly elevated in the meiocyte (Fig. 1d). The higher abundance of Pol V pathway components in the meiocyte, together with a high siRNA load (Fig. 1d), potentially enable siRNAs to direct methylation beyond loci with perfect sequence homology.

### AGO4/5/6/9-bound sRNAs are predominantly derived from *pol-iv*-dependent siRNA clusters

**Fig. 2:**
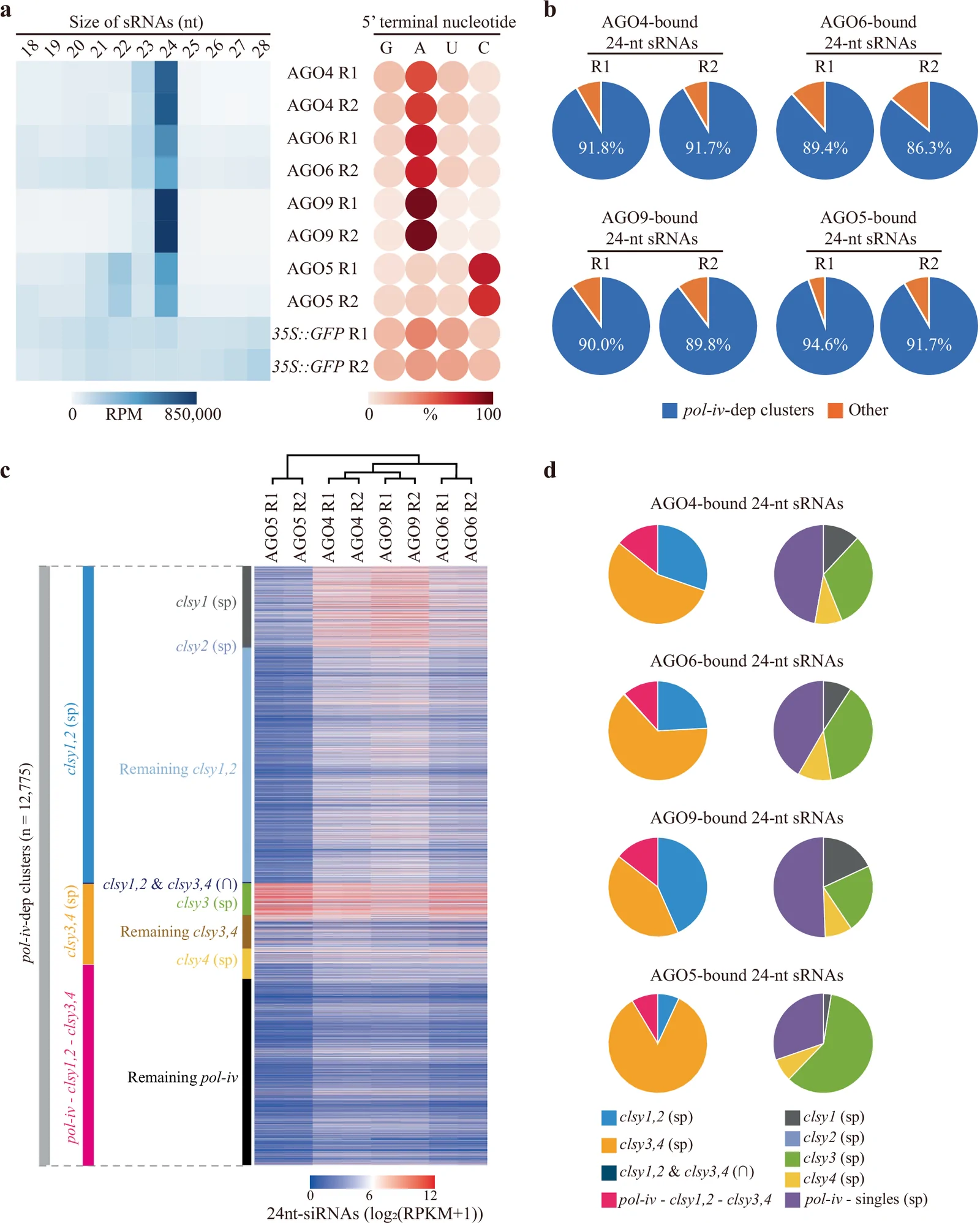
The majority of AGO4/5/6/9-bound sRNAs are mapped to *pol-iv*- and *clsy3,4*-dependent clusters. **a** Heatmap displaying normalized abundance (Reads per million, RPM) of sRNAs (18-28 nt) loaded into AGO4, AGO5, AGO6, and AGO9 (left panel) and their 5′ terminal nucleotide composition (right panel). R, biological replicates; RPM, reads per million mapped reads. **b** Pie charts illustrate the percentages of AGO4-, AGO5-, AGO6- and AGO9-bound 24-nt sRNAs (from two biological replicates) that align perfectly with *pol-iv*-dependent siRNA clusters. **c** Heatmap showing normalized abundance of AGO4-, AGO5-, AGO6- and AGO9-bound 24-nt across individual loci of *pol-iv*- or *clsy*- dependent clusters (clusters were defined in ref.^5^) Sp, specific; ∩, intersection; *pol-iv* – *clsy1,2* – *clsy3,4* indicates *pol-iv*-dependent clusters that are neither *clsy1,2*- nor *clsy3,4*-dependent. RPKM, reads per kilobase of transcript, per million mapped reads. **d** Pie charts displaying the proportions of AGO4/5/6/9-bound 24-nt sRNAs derived from distinct categories of *pol-iv*- or *clsy*- dependent sRNA clusters.

To determine the sRNA populations incorporated into AGO4, AGO5, AGO6, and AGO9, we performed small RNA immunoprecipitation followed by high-throughput sequencing (RIP-seq) using flower buds from corresponding reporter lines (Supplementary Table 1). Consistent with previous findings^26,50,51^, AGO4, AGO6, and AGO9 predominantly associate with 24-nt siRNAs, whereas AGO5 binds to 21-, 22-, and 24-nt sRNAs, with 24-nt being the most abundant (42.4% of all AGO5-bound sRNAs) (Fig. 2a). Analysis of the 5′ terminal nucleotide preference revealed that AGO4, AGO6, and AGO9 preferentially load sRNAs with 5’A (63.1%, 74.3% and 77.6%, respectively), while AGO5 favors sRNAs with a 5’C (72.7%) (Fig. 2a); as a control, sRNAs pulled down by GFP from *pro35S::GFP* transgenic plants displayed no discernible size or 5′ nucleotide bias (Fig. 2a). These analyzes confirmed the robustness of our data.

Mapping AGO4/5/6/9-bound sRNAs to the *Arabidopsis* reference genome showed that the majority (~90%) of AGO4/5/6/9-bound 24-nt sRNAs originate from *pol-iv*-dependent siRNA clusters (defined in ref.^5^) in flower buds (Fig. 2b). Additionally, more than 60% and 80% of AGO5-bound 21-nt and 22-nt sRNAs, respectively, also map to *pol-iv*-dependent siRNAs clusters (Supplementary Fig. 2a). These findings suggest that the biogenesis of siRNAs incorporated into AGO4, AGO5, AGO6 or AGO9 is largely dependent on Pol IV.

To investigate whether AGO4-, AGO5-, AGO6-, and AGO9-bound siRNAs originate from distinct regions within *pol-iv*-dependent sRNA clusters, we quantified the abundance of 24-nt siRNAs bound to each AGO at individual loci (Fig. 2c). Hierarchical analysis revealed that AGO4-, AGO6- and AGO9-bound siRNAs clustered together, indicative of similar distribution patterns, whereas AGO5-bound siRNAs exhibited a different pattern (Fig. 2c). Compared to AGO4/6/9-bound siRNAs, AGO5-bound siRNAs accumulated more at *clsy3*- and *clsy3,4*-dependent clusters and less at *clsy1,2*-dependent clusters (Fig. 2d). These results suggest that while AGO4, AGO6, and AGO9 bind Pol IV-siRNAs from similar genomic loci, AGO5 preferentially loads siRNAs from a subset of these loci that are largely dependent on CLSY3. Notably, most AGO5-bound 21- and 22-nt siRNAs also mapped to *clsy3-* or *clsy3,4*-dependent sRNAs clusters (Supplementary Fig. 2b). Furthermore, the abundance values of AGO5-bound 21-, 22-, and 24-nt siRNAs at *clsy3*-dep clusters were highly correlated (Supplementary Fig. 2c), supporting the hypothesis that the production of AGO5-bound 21/22-nt siRNAs, like AGO5-bound 24-nt siRNAs, relies on CLSY3 and Pol IV.

### Pol IV and CLSY3 are essential for the biogenesis of AGO5-bound 21-, 22-, and 24-nt siRNAs

**Fig. 3:**
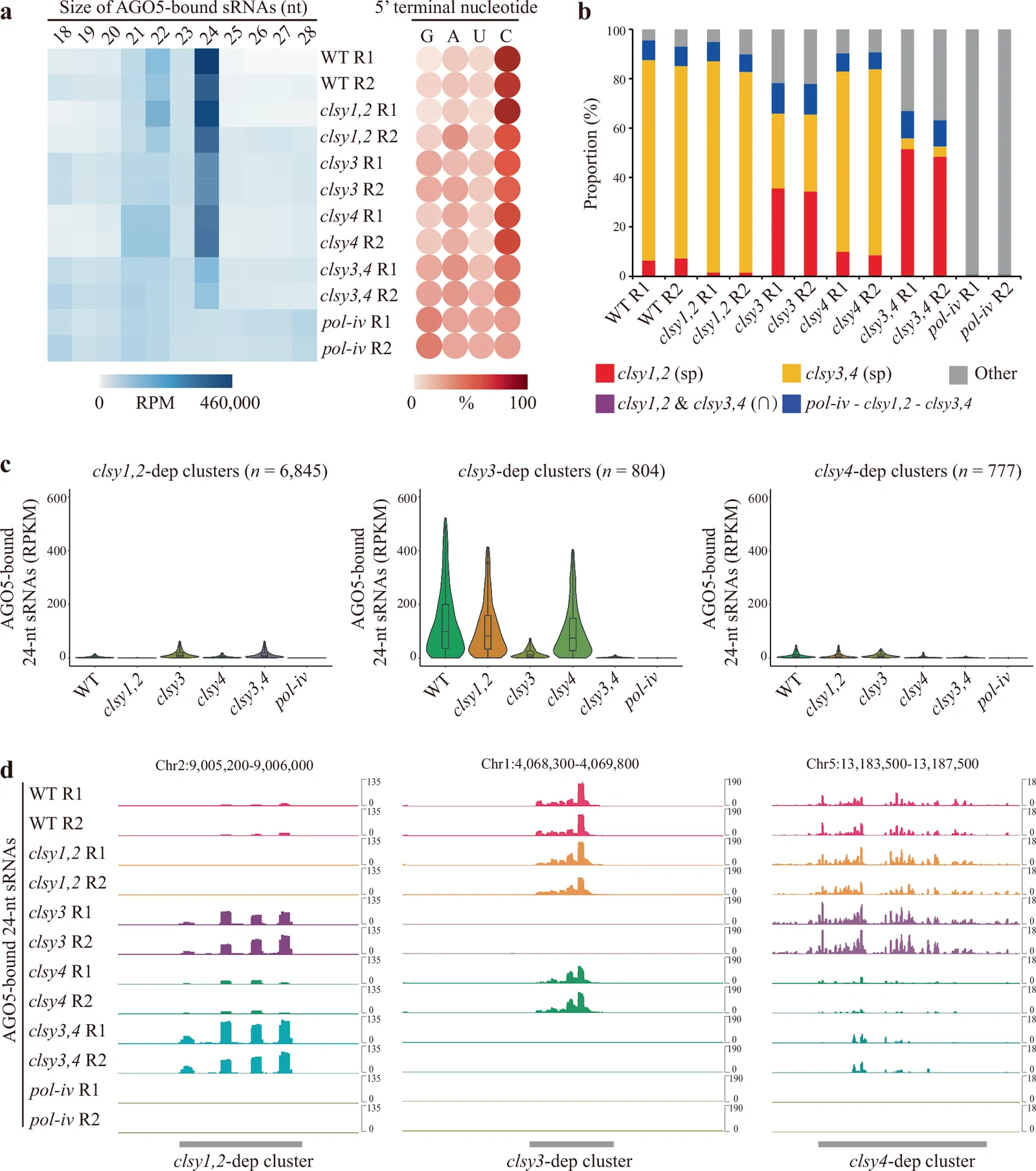
Biogenesis of AGO5-bound siRNAs requires Pol IV and CLSY3. **a** Heatmap showing the normalized abundance of each size (18-28 nt) of AGO5-bound sRNAs in different genetic backgrounds (left panel) with the 5′ terminal nucleotide composition of these sRNAs shown in the right panel. R, biological replicate. **b** Bar plots showing the proportions of AGO5-bound 24-nt sRNAs derived from various categories of *clsy*-dependent clusters (as used in Fig. 2c) relative to the total number derived from *pol-iv*-dependent clusters. **c** Boxplots representing the levels of AGO5-bound 24-nt sRNAs at indicated *clsy*-dependent clusters across various mutant backgrounds. Box plots show the median (center line), interquartile range (box), and 1.5 × IQR whiskers. **d** Genome browser snapshots displaying the abundance of AGO5-bound 24-nt sRNAs at representative loci from *clsy*-dependent clusters in indicated genotypes.

Given that AGO5 is cytoplasmically expressed and evolutionally closest to AGO1, it is unexpected that AGO5-bound 21-, 22- and 24-nt siRNAs are predominantly derived from *pol-iv*-dependent sRNA clusters. To determine whether the biogenesis of AGO5-bound siRNA depends on Pol IV, we crossed the AGO5 reporter line (*pAGO5::Venus-AGO5*) with *pol-iv* to generate *pAGO5::Venus-AGO5* transgenic plants in the *pol-iv* mutant background (*pol-iv, pAGO5::Venus-AGO5*). We then performed AGO5 RIP-seq and analyzed the loaded sRNAs. Our results showed a sharp decrease in the abundance of AGO5-bound 21-, 22-, and 24-nt sRNAs in *pol-iv* compared to WT (Fig. 3a). Additionally, the 5’ C preference for AGO5-bound sRNAs was also abolished (Fig. 3a). To exclude the possibility that this reduction results from decreased AGO5 abundance in the *pol-iv* background, we examined *pAGO5::Venus-AGO5 pol-iv* plants by confocal microscopy and observed comparable AGO5 signals in meiocytes relative to *pAGO5::Venus-AGO5* (Supplementary Fig. 3a). These results demonstrate that AGO5-bound siRNAs are largely produced by Pol IV.

Since the vast majority of AGO5-bound siRNAs mapped to *clsy3-* and *clsy3,4*-dependent clusters (Fig. 2d, and Supplementary Fig. 2b), we introduced *pAGO5::Venus-AGO5* into a series of *clsy* mutants, including *clsy1,2* double, *clsy3* single, *clsy4* single, and *clsy3,4* double mutants. The overall abundance of AGO5-bound sRNAs, particularly 24-nt siRNAs, was greatly reduced in *clsy3* and *clsy3,4* mutants but remained largely unchanged in the *clsy1,2* mutant (Fig. 3a). Consistently, the loss of AGO5-bound sRNAs in *clsy3* and *clsy3,4* mutants also resulted in a noticeable decrease in the 5′C preference (Fig. 3a). Importantly, confocal imaging revealed comparable AGO5 levels in meiocytes of *clsy3* mutants and control plants (Supplementary Fig. 3a), excluding reduced AGO5 abundance as a cause of the observed sRNA loss. Together, these results show that AGO5-bound 24-nt siRNAs are primarily dependent on CLSY3 (and to a lesser extent, CLSY4). Consistent with the known ability of CLSY3-dependent siRNAs to target genic loci in meiocytes with reduced sequence complementarity, AGO5-bound 24-nt siRNAs, like those associated with AGO4/6/9, have the capacity to target MetGenes with up to three mismatches (Supplementary Fig. 3b).

We further examined AGO5-bound siRNAs at different *clsy*-dependent clusters (Fig. 3b, c, and Supplementary Fig. 3c–g). Consistent with the above findings, *clsy3*-dependent clusters showed a substantially higher enrichment of 24-nt AGO5-bound siRNAs, compared to *clsy1,2*- and *clsy4*-dependent clusters (Fig. 3b–d). This enrichment was significantly reduced in the *clsy3* mutant, confirming its dependency on CLSY3 (Fig. 3c, d). In the *clsy3,4* double mutant, AGO5-bound 24-nt siRNAs were further depleted (Fig. 3c, d, and Supplementary Fig. 3c), indicating that CLSY4 also plays a minor and partially redundant role in driving the production of these siRNAs. AGO5-bound 21- and 22-nt siRNAs displayed similar trends across these *clsy* mutants (Supplementary Fig. 3d–g). Taken together, these results establish that the biogenesis of AGO5-bound siRNAs is mediated by Pol IV and CLSY proteins, with CLSY3 playing a predominant role.

### Dosage-dependent role of AGO4, AGO6 and AGO9 in tapetal siRNA-induced DNA methylation in meiocytes

**Fig. 4:**
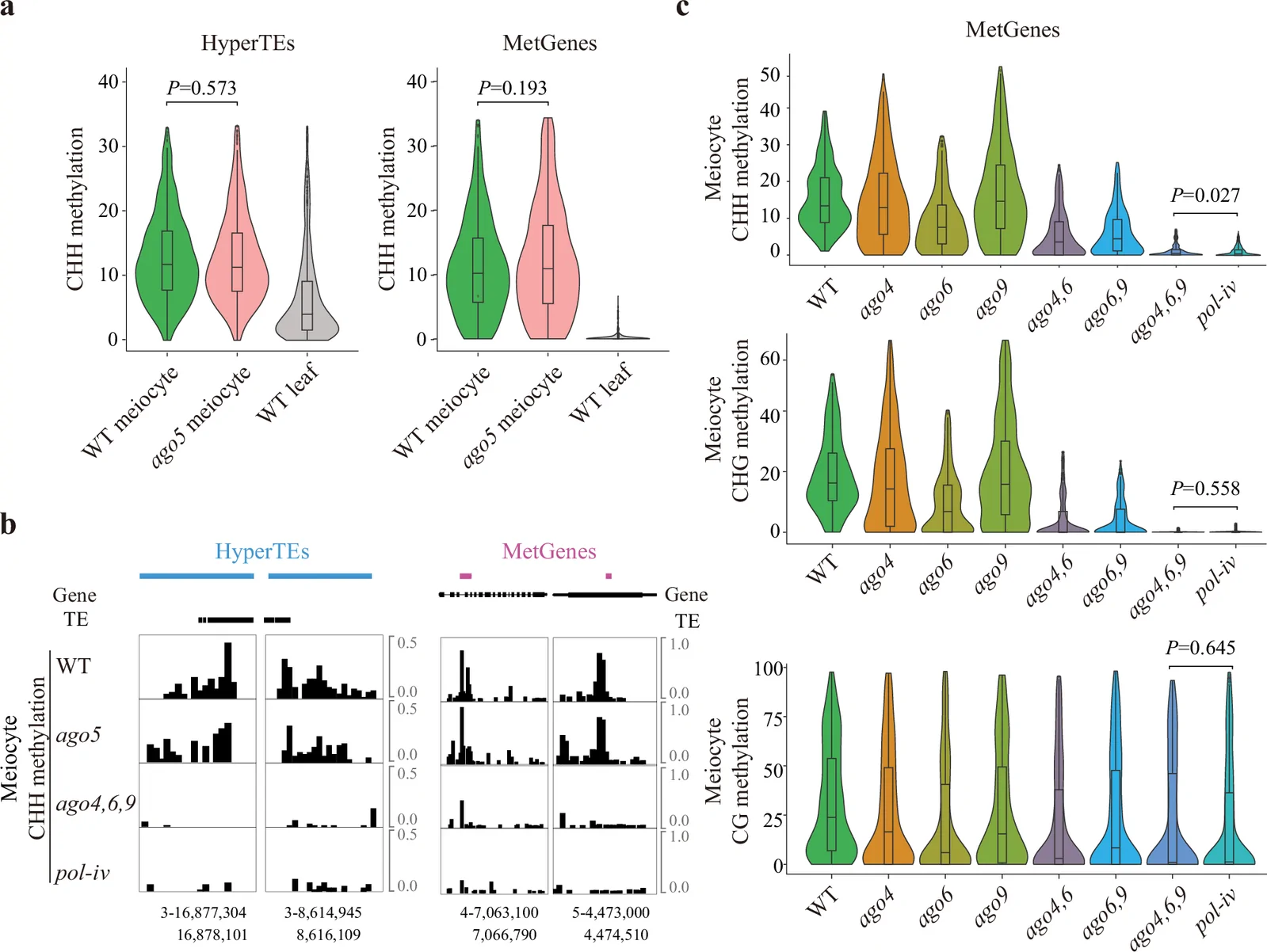
AGO4/6/9, but not AGO5, mediate de novo methylation in meiocytes. **a** Boxplots depicting CHH methylation levels of HyperTEs (*n* = 797) and MetGenes (*n* = 469) in the meiocytes of WT and *ago5* mutant, and WT leaf. **b** Genome browser snapshots showing the CHH methylation at representative HyperTE (blue color) or MetGene (pink color) loci in WT, *ago5*, *ago4,6,9* and *pol-iv* meiocytes. **c** Boxplots displaying DNA methylation levels at MetGenes (*n* = 469) in meiocytes from plants of indicated genotypes. Note that the WT controls in (**a**) and (**c**) were from different plants grown side by side with the corresponding mutant plants in the same panel. Consistent with the knowledge that CHH methylation is influenced by the environment^53^, these WT controls show slightly different methylation levels. For all box plots unless otherwise indicated, the center line indicates the median, the box indicates the interquartile range (IQR), the whiskers extend to 1.5 × IQR, and *P* values were calculated using a two-sided Kolmogorov–Smirnov test.

Since AGO5 predominantly associates with Pol IV-derived siRNAs, we tested its role in establishing de novo DNA methylation in the male meiocyte. We performed whole-genome bisulfite sequencing (BS-seq) on meiocytes isolated from wild-type (WT) and *ago5* mutant plants (Supplementary Table 2). Consistent with our previous findings^43,44^, both MetGene and HyperTE loci exhibited significant CHH hypermethylation in WT meiocytes compared to WT somatic tissue (Fig. 4a, b). Methylation levels at both sets of loci remained unaffected in *ago5* meiocyte (Fig. 4a, b), indicating that the loss of AGO5 alone has a minimal impact on the methylation reprogramming in meiocytes. This is consistent with the lack of AGO5 signal in the nucleus (Fig. 1b).

To investigate the role of AGO4, AGO6, and AGO9 in establishing meiocyte DNA methylation, we performed BS-seq on meiocytes isolated from *ago4*, *ago6*, *ago9*, *ago4*,*6*, *ago6*,*9*, and *ago4*,*6*,*9* mutants (Supplementary Table 2). We observed that single mutations caused slight DNA methylation reduction at MetGenes (Fig. 4c). Among these, *ago6* single mutant showed a stronger effect than *ago4* or *ago9* (Fig. 4c). DNA methylation was further reduced in *ago4,6* and *ago6,9* double mutants, and the *ago4,6,9* triple mutant completely abolished the hypermethylation at both sets of loci, reducing methylation to levels observed in *pol-iv* mutant meiocyte (Fig. 4b, c). These findings suggest semi-redundancy between AGO4, AGO6, and AGO9 and support our model in which the high overall dosage of AGO proteins in meiocytes facilitates the broad targeting ability of siRNAs (Fig. 1d). Similarly, DNA methylation at HyperTEs was reduced by *ago4*, *ago6* and *ago9* compound mutations (Supplementary Fig. 4). Together, these results demonstrate that tapetal siRNAs-induced hypermethylation in the meiocyte depends on the dosage of AGO4, AGO6, and AGO9 proteins. The comparable DNA methylation levels between *ago4,6,9* triple and *pol-iv* mutants (Fig. 4c) suggest that AGO5 does not contribute to DNA methylation establishment in meiocytes.

### AGO5 and AGO4/6/9 work synergistically to regulate gene expression in meiocytes

**Fig. 5:**
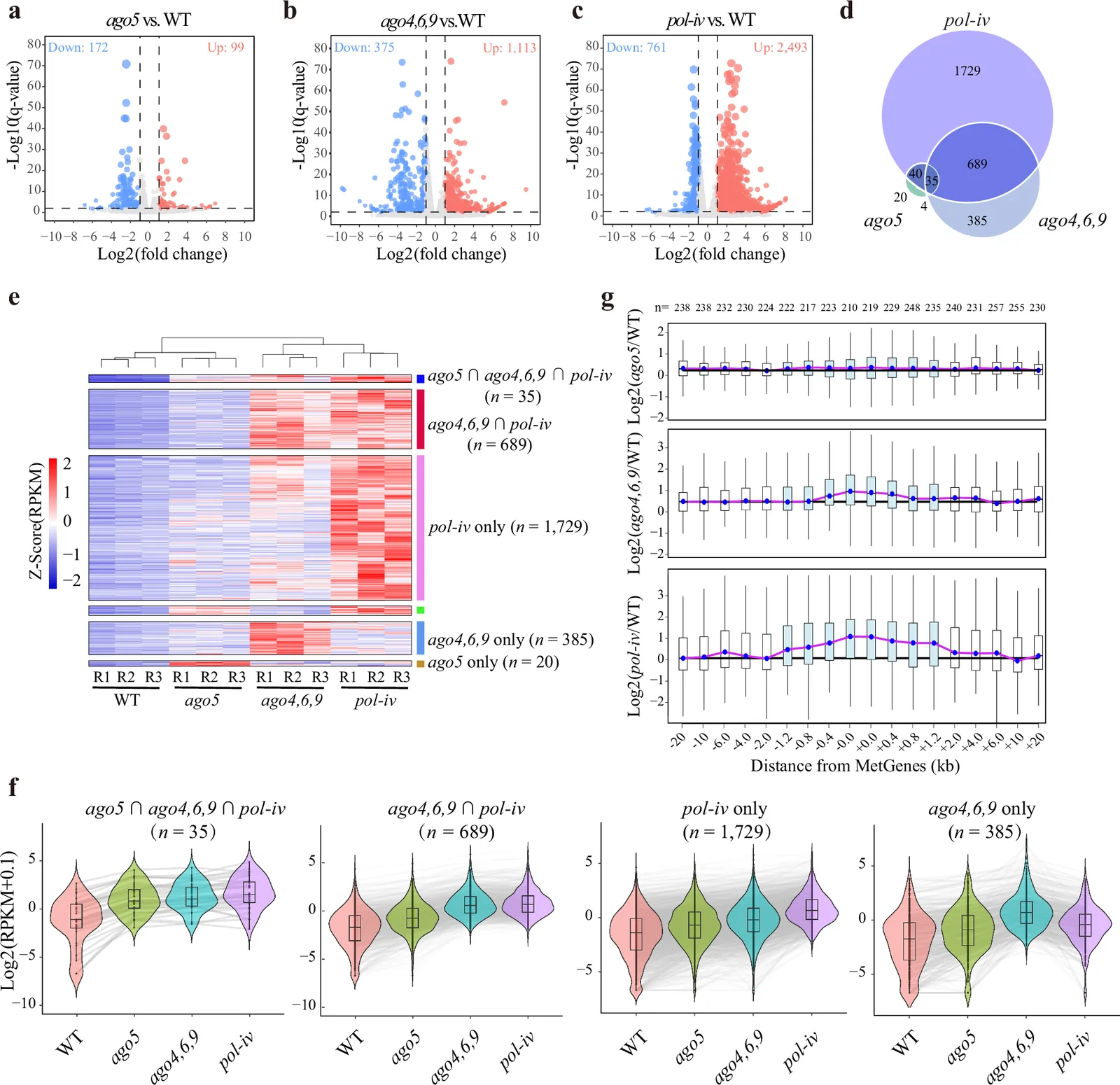
AGO5 and AGO4/6/9 play synergistic roles to regulate gene expression in meiocytes. **a**–**c** Volcano plots illustrating the numbers of differentially expressed genes (DEGs) identified in the meiocyte of *ago5*, *ago4,6,9*, and *pol-iv* compared to WT. DEGs, log2 (fold change) ≥1 and *q* < 0.01. **d** Venn diagram displaying the overlap of up-regulated genes found in the meiocyte of *pol-iv*, *ago4,6,9*, and *ago5*. **e** Heatmap showing the expression levels of genes categorized into different groups in the meiocyte of WT, *ago5*, *ago4,6,9*, and *pol-iv*, with RNA-seq data from three biological replicates (R1-3). Sp, specific; ∩, intersection. **f** Boxplots indicating the expression levels of different sets of genes in WT, *ago5*, *ago4,6,9*, and *pol-iv* meiocytes. The number of genes in each group is showed in (**e**). Boxes show the interquartile range (IQR), with the center line indicating the median and whiskers extending to 1.5 × IQR. **g** Expression changes of genes located within the flanking 20 kb regions of MetGenes in the meiocyte of *ago5* vs. WT (upper panel), *ago4,6,9* vs. WT (middle panel), and *pol-iv* vs. WT (lower panel). The number of genes in each box plot is indicated at the top of the panel. The box plots are formatted as described in (**f**).

We next investigated the impact of these meiocyte-enriched AGOs on transcription by performing RNA-sequencing (RNA-seq) on meiocytes isolated from WT, *ago5, ago4,6,9*, and *pol-iv* mutants (Supplementary Table 3). We identified 271, 1488, and 3254 differentially expressed genes (DEGs) in *ago5, ago4,6,9*, and *pol-iv* meiocytes, respectively, in comparison to WT meiocytes (Fig. 5a–c, Supplementary Fig. 5a, b, and Supplementary Data 1). These DEGs exhibited significantly higher levels of AGO-associated siRNAs than non-DEGs (Supplementary Fig. 5c), suggesting that at least a subset of these genes are direct targets of siRNA-mediated regulation.

Consistent with the RdDM pathway’s prevalent role in transcriptional repression^52^, the majority (approximately 75%) of DEGs were upregulated in *ago4,6,9* and *pol-iv* mutants (Fig. 5b, c). Moreover, upregulated DEGs in *ago4,6,9* and *pol-iv* meiocytes significantly overlapped with MetGenes, whereas *ago5* DEGs and downregulated DEGs in *ago4,6,9* or *pol-iv* meiocytes did not show significant overlap (Supplementary Fig. 5d). Nevertheless, the overlap between upregulated DEGs and MetGenes remained relatively small (~2%; Supplementary Fig. 5d), suggesting that most transcriptional changes are likely indirect consequences rather than direct effects of DNA methylation loss. Consistent with AGO4, AGO6, AGO9, and Pol IV functioning in the same de novo methylation pathway, 65% (724/1113) of upregulated and 27% (100/375) of downregulated genes in *ago4,6,9* meiocytes overlapped with those showing corresponding changes in *pol-iv* meiocytes (Fig. 5d, and Supplementary Fig. 5e). Together, these results indicate that the Pol IV–AGO4/6/9 pathway exerts broad effects on meiocyte gene expression, despite directly methylating only a limited subset of genes.

Previously, we showed that the de novo methylation pathway promotes male meiosis, as the *drm1 drm2* double mutant produces an increased proportion of triads at the end of meiosis compared to WT (7% in *drm1 drm2* versus 0% in WT)^44^. This phenotype was shown to result from defective splicing of the key meiotic gene *MULTIPOLAR SPINDLE 1* (*MPS1*; also known as *PUTATIVE RECOMBINATION INITIATION DEFECTS 2*, *PRD2*), in which loss of DNA methylation at the last intron causes increased intron retention, generating a premature stop codon and reducing functional *MPS1* transcript levels^44^. Consistently, we detected elevated retention of the last *MPS1* intron in both *ago4,6,9* triple mutant and *pol-iv* mutant meiocyte RNA-seq data, which was further confirmed by RT-PCR (Supplementary Fig. 6a). Correspondingly, both *ago4,6,9* triple mutant and *pol-iv* mutant also exhibited elevated frequencies of meiotic triads relative to WT (Supplementary Fig. 6b, and Supplementary Table 4). The penetrance of this phenotype was modestly lower than previously reported for *drm1 drm2* mutant (4% versus 7%), likely reflecting differences in growth conditions, which are known to influence RdDM activities^53–56^.

Although fewer genes were misregulated in *ago5* meiocytes compared to *pol-iv* and *ago4,6,9* meiocytes, a substantial proportion of both upregulated (76%) and downregulated genes (32%) in *ago5* meiocytes overlapped with those with corresponding changes in *pol-iv* meiocytes (Fig. 5d, and Supplementary Fig. 5e), consistent with our finding that AGO5 binds Pol IV-derived siRNAs. Additionally, 39% of upregulated genes in *ago5* meiocytes overlapped with those upregulated in *ago4,6,9* meiocytes (Fig. 5d), suggesting that AGO5 may function in partial redundancy with AGO4/6/9 in regulating meiocyte gene expression. Supporting this idea, genes upregulated in *ago4,6,9* or *pol-iv* meiocytes often showed a modest transcriptional increase in *ago5* meiocytes (Fig. 5e, f). However, because AGO5 does not contribute detectably to DNA methylation and is predominantly localized in the meiocyte cytoplasm, these observations suggest that AGO5 modulates gene expression through an alternative mechanism, potentially PTGS. The *ago5* mutant nevertheless exhibited a modest but significant increase in triad formation together with elevated *MPS1* mis-splicing (Supplementary Fig. 6a, b, and Supplementary Table 4), suggesting that AGO5 partially converges with meiotic regulatory pathways controlled by Pol IV and AGO4/6/9.

Notably, a large number (1729) of DEGs were categorized as being specifically upregulated in *pol-iv* mutant meiocytes (Fig. 5d). These transcripts tend to be slightly elevated in *ago5* and *ago4,6,9* mutants, suggesting that AGO5 and AGO4,6,9 have distinct, but synergistic roles in gene regulation in meiocytes (Fig. 5e, f). In addition, a subset of genes upregulated in *ago4,6,9* mutants did not exhibit significant expression changes in *pol-iv* or *ago5* mutants (Fig. 5e). This aligns with the known flexibility of the siRNA pathways in adapting to disruptions by utilizing alternative non-canonical component proteins, as previously demonstrated for DCL proteins, Pol IV and Pol II, and AGOs^57,58^.

Since DNA methylation of MetGenes was abolished in *ago4,6,9* and *pol-iv* meiocytes, we next evaluated how MetGene-targeted gene expression was affected in these mutants. We assessed expression changes in genes located within 20-kilobase (kb) regions flanking MetGenes. Consistent with DNA methylation patterns, genes located near MetGenes (<1.2 kb) exhibited a clear trend of transcriptional upregulation in *pol-iv* and *ago4,6,9* meiocytes, but not in *ago5* meiocytes (Fig. 5g). These results align with our previous study^44^ and further support that DNA methylation at MetGenes plays an essential role in transcriptional regulation in meiocytes.

### Distinct but intersecting roles of AGO5 and AGO4/6/9 in transposon silencing in meiocytes

**Fig. 6:**
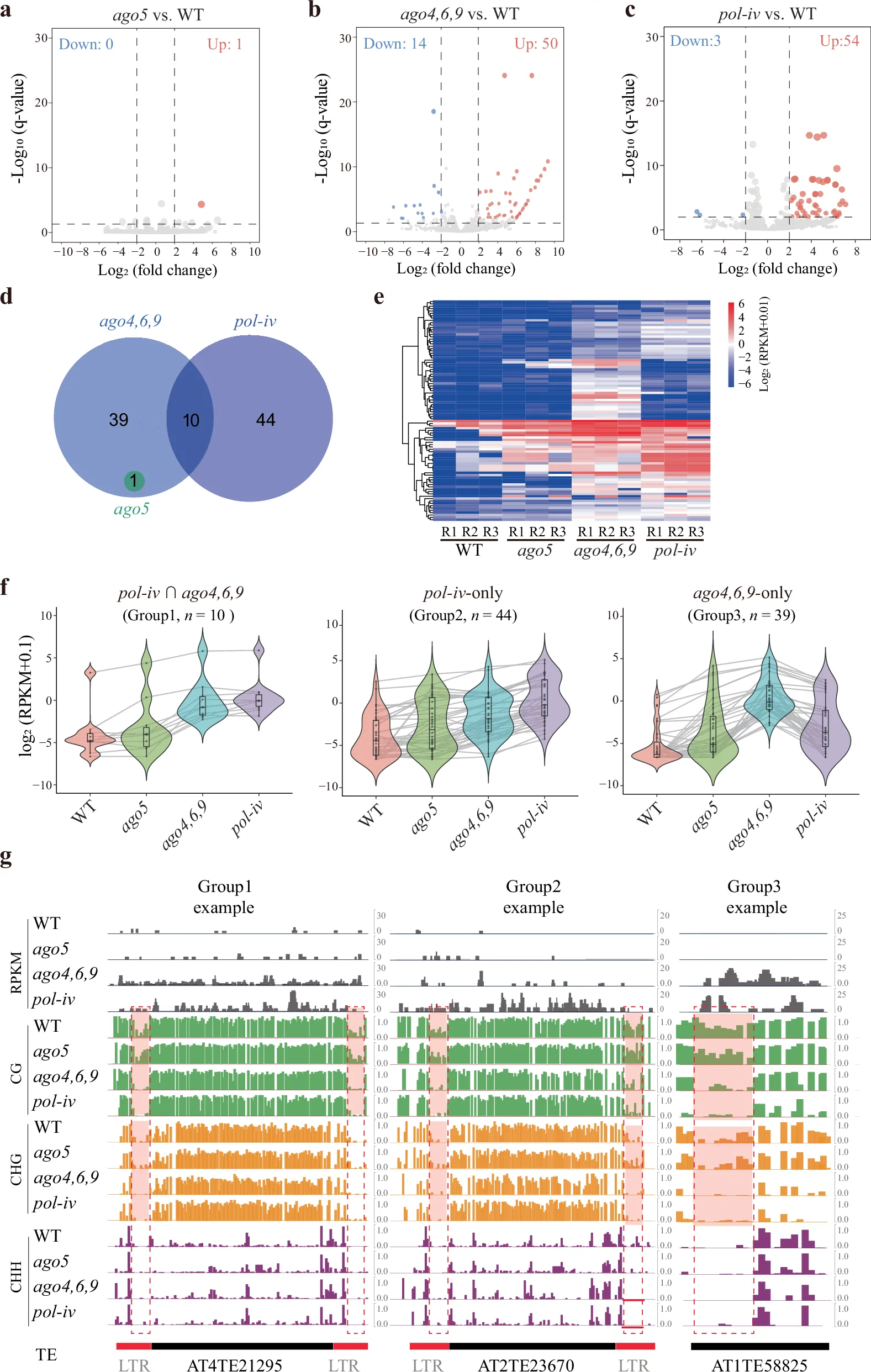
Distinct yet intersecting roles of AGO5 and AGO4/6/9 in silencing TEs in meiocytes. **a**–**c** Volcano plots showing the number of differentially expressed TEs (DETEs) identified in the meiocytes of *ago5*, *ago4,6,9*, and *pol-iv* compared to WT. DETEs, log_2_ (fold change) ≥2 and *q* < 0.01. **d** Venn diagram illustrating the overlap of up-regulated TEs identified in the meiocytes of *pol-iv*, *ago4,6,9*, and *ago5*. **e** Heatmap showing the expression level of TEs that are upregulated in the meiocytes of *pol-iv*, *ago4,6,9*, or *ago5*, compared to WT. **f** Boxplots showing the expression levels of different groups of TEs (as categorized in **d**) across different genotypes. The sample sizes of Groups 1, 2 and 3 are 10, 44 and 39, respectively. Boxes show the interquartile range (IQR), with the center line indicating the median and whiskers extending to 1.5 × IQR. **g** Transcription and DNA methylation at examples of different groups of TEs (as displayed in **f**). Red dashed boxes indicate differentially methylated regions between *ago4,6,9* and *pol-iv* versus WT, with those displaying methylation differences in a particular sequence context shaded.

To investigate the involvement of AGO5, AGO4/6/9 and Pol IV in TE regulation in meiocytes, we compared TE transcription in these mutants to that in WT meiocytes. While only one differentially expressed TE (DETE) was identified in *ago5* meiocytes, 64 and 57 DETEs were detected in *ago4,6,9* and *pol-iv* meiocytes (Supplementary Data 2), respectively, with the majority being upregulated (Fig. 6a–c). Upregulated TEs are associated with significantly higher levels of AGO4/6/9-bound siRNAs, compared to downregulated or non-differentially expressed TEs (Supplementary Fig. 7a), consistent with the established role of RdDM in TE repression^43^. TE family enrichment analysis revealed that the AtGP1 family was enriched among upregulated TEs in *pol-iv* meiocytes, whereas both the META1 and AtGP1 families were highly represented among upregulated TEs in *ago4,6,9* meiocytes (Supplementary Fig. 7b, c). This is consistent with our previous finding that an AtGP1 Gypsy retrotransposon is specifically derepressed in *rdr2* single and *drm1 drm2* double mutant male germlines^43^. Our data demonstrate that AGO4/6/9 and Pol IV are key regulators of TE silencing in meiocytes.

Among the 50 upregulated TEs in *ago4,6,9* mutant meiocytes, 10 (20%) were also substantially upregulated in the *pol-iv* mutant (Fig. 6d, e, and Supplementary Fig. 7d), indicating that their silencing depends on AGO4/6/9- and Pol-IV-mediated DNA methylation. Indeed, these transposons, primarily from the AtGP1 family, showed significant loss of DNA methylation at their long terminal repeat (LTR) sequences in *ago4,6,9* and *pol-iv* mutant meiocytes (but not in *ago5* mutant meiocytes) (Fig. 6f, g). In contrast, the remaining 40 (80%) upregulated TEs in *ago4,6,9* mutant meiocytes were not upregulated by more than fourfold in *pol-iv* mutant meiocytes (Fig. 6d). Most (34 out of 40, 85%) of these exhibited mild upregulation in *pol-iv* meiocytes, such as *AT1TE58825* (Fig. 6f, g), whereas some others remain unaffected in *pol-iv* mutant (Fig. 6f). This result is consistent with the strong flexibility of siRNA pathways^57,58^ mentioned in the previous section and suggests that AGO4/6/9 also participate in Pol IV-independent RNA interference pathways to maintain TE silencing in the germline.

Additionally, 44 TEs were categorized as being only activated in the *pol-iv* mutant (Fig. 6d). A closer look at the RNA-seq data revealed that many (42 of 44, 95%) of these TEs nonetheless exhibited mild increases of transcription in *ago4,6,9* mutant meiocytes, exemplified by *AT2TE23670* from the AtGP1 family (Fig. 6e, f). Consistent with this derepression, the LTR regions of *AT2TE23670* showed reduced DNA methylation in both *ago4,6,9* and *pol-iv* mutants (Fig. 6g), and its transcriptional activation was further confirmed using an LTR-YFP reporter (Supplementary Fig. 8). Notably, 33 of these 44 (75%) TEs also showed slight upregulation in the *ago5* mutant (Fig. 6e, f). Similarly, for the 40 TEs upregulated exclusively in *ago4,6,9* meiocytes, 29 (72.5%) of them also exhibited a slight elevation in *ago5* (Fig. 6e, f). Therefore, AGO5 also contributes to TE silencing in meiocytes, likely through PTGS. Together, these results indicate that Pol IV-derived siRNAs mediate TE repression in the meiocytes through synergistic AGO5-mediated PTGS and AGO4/6/9-mediated DNA methylation.

## Discussion

Our study reveals that *Arabidopsis* AGO4, AGO5, AGO6, and AGO9 are highly expressed in meiocytes and associate with Pol IV-dependent siRNAs. We also show that Pol V is strongly enriched in meiocytes, whereas Pol IV is absent in meiocytes but highly expressed in the tapetum. Genetic and transcriptomic analyzes demonstrate that AGO4, AGO6, and AGO9 act in a dosage-dependent manner to mediate DNA methylation at MetGenes, thereby regulating gene transcription in meiocytes. Collectively, these results elucidate three key mechanisms by which tapetal siRNAs methylate genic regions with imperfect sequence homology: (1) elevated Pol IV pathway activity in the tapetum ensures the production of abundant siRNAs; (2) highly expressed Pol V pathway components (inc. AGOs and Pol V) compensate for reduced affinity due to siRNA-scaffold mismatches and allow broad targeting; (3) Pol IV absence in meiocytes prevents the generation of perfect-matching siRNAs from MetGenes, restricting target expansion (Fig. 1d).

Unexpectedly, we find AGO5 binds 21, 22, and 24-nt siRNAs that are predominantly dependent on Pol IV and CLSY3 for their production. Given that AGO5 and CLSY3 are exclusively expressed in the meiocyte and tapetum, respectively, during the meiotic stage in the anther, and that the production of tapetal siRNAs requires CLSY3, this indicates that tapetal siRNAs also bind AGO5 in the meiocyte, in addition to AGO4, 6 and 9. However, our data show that AGO5 does not mediate DNA methylation in meiocytes, because: (1) AGO5 is localized in the meiocyte cytoplasm (Fig. 1b); (2) *ago5* mutants show no significant hypomethylation (Fig. 4a) and (3) *ago4,6,9* triple mutants exhibit comparable methylation loss to *pol-iv* mutants (Fig. 4c). A previous study reported AGO5 association with 21-22 nt siRNAs and its involvement in DNA methylation in sperm cells^26^. Together, these observations suggest AGO5 undergoes a stage-specific functional shift between meiocytes and sperm cells, highlighting functional diversification of AGO5 during male germline development.

Interestingly, these AGO4/5/6/9-bound, Pol IV-derived siRNAs show striking similarities with phasiRNAs in monocots. These siRNAs are produced by the tapetum and transported into meiocytes during meiotic prophase I, resembling 24-nt phasiRNAs in maize^34,43^. Second, their source loci (HyperTEs) are hypermethylated, similar to the source loci of 24-nt phasiRNAs in maize^59^. Like phasiRNAs, these siRNAs are important for male fertility, as both *ago4,6,9* and *ago5* mutants display significant meiotic defects (Supplementary Table 4). Finally, the AGO5 homolog in rice is also meiocyte-specific and is associated with phasiRNAs^40,60,61^. These similarities suggest that Pol IV-derived siRNAs may functionally substitute for phasiRNAs in *Arabidopsis*. This idea is highly plausible, given the well-documented plasticity of siRNA pathways^57,58^, including this study. Given its simple genome and tractable genetics, *Arabidopsis* thus serves as an excellent model for investigating phasiRNA mechanisms. Our findings indicate that tapetal siRNAs regulate gene expression and TE silencing in meiocytes via distinct yet complementary pathways mediated by AGO5 and AGO4/6/9. Exploring these mechanisms in monocot crops could deepen our understanding of phasiRNA function and facilitate their application in agriculture. Given their crucial role in fertility, particularly in response to environmental cues, this knowledge could be leveraged to develop environment-sensitive male sterility lines for hybrid seed production.

## Methods

### Plant material

Plants used in this study (*Arabidopsis thaliana* Col-0) were grown under 16 h light/8 h dark in a growth chamber (21 °C, 70% humidity). The following published mutants were used in this study: *ago4-5*^62^, *ago9-2*^63^, *ago5-1*^64^*, nrpd1-3*^65^ *(pol-iv), clsy1-7, clsy2-1*, *clsy3-1*, *clsy4-1*^5^. The *ago4-4 ago6-2* (*ago4*,*6*) double*, ago6-2 ago9-1* (*ago6*,*9*) double, and *ago4-4 ago6-2 ago9-1* (*ago4*,*6*,*9*) triple mutants were obtained from Wang et al.^49^. The *clsy1,2* and *clsy3,4* double mutants were generated from our previous study^43^. Transgenic plants were obtained by Agrobacterium-mediated floral dip. Individual *Venus-AGO* constructs (AGO1-AGO10), driven by their native promoters, were transformed into Col-0 wild-type plants. For these, two independent T1 lines were used for cytometric analysis, and T2 transgenic plants of AGO4/5/6/9 reporter lines were used for AGO immunoprecipitation assays. The *pAGO5::Venus-AGO5* constructs were transformed into, respectively, *pol-iv*, *clsy1,2*, *clsy3*, *clsy4*, and *clsy3,4* mutant backgrounds by crossing *pAGO5::Venus-AGO5* (Col-0) with the corresponding mutant. Both *pNRPD1::NRPD1-GFP* and *pNRPE1::NRPE1-GFP* constructs were transformed into plants expressing *pRPS5A::RFP-TUA5*^66^ (Col-0 background), which labels the microtubule cytoskeleton, via the Agrobacterium-mediated floral dip method. T1 plants were recovered and used for microscopic examination. The *GP1*_*LTR*_*::YFP* reporter line (Col-0 background)^43^ was crossed into *ago5*, *ago4,6,9* and *pol-iv* mutant backgrounds.

### Isolation of meiocyte

*Arabidopsis* male meiocytes (meiotic prophase I) were isolated as described previously^44^. Briefly, stage 9 flower buds were collected and gently squeezed between a glass slide and a coverslip. The released meiocytes were examined carefully under a microscope. Clean meiocytes free from any somatic-cell debris were transferred to a new slide with capillary glass pipettes, washed with 1× PBS buffer three times and frozen in liquid nitrogen. RNA and DNA were extracted for subsequent library preparation.

### Plasmid construction

For the AGO (AGO1/2/3/4/5/6/10) reporter constructs, approximately 2 kb upstream of each *AGO* gene was cloned as the promoter and each *AGO* gDNA sequence (including introns) was amplified using the primers listed in Supplementary Data 3. The PCR products were first cloned into the donor vector *pK7m34GW*^67^, followed by cloning into the destination vector *pGWB13-Bar*^44^ using the MultiSite Gateway method (Invitrogen, cat. no. 11791-020). For AGO7 *and* AGO9 reporter constructs, approximately 2 kb upstream of each gene was cloned as the promoter. The Venus tag and each *AGO* gDNA sequence (incl. introns) were amplified using the primers listed in Supplementary Data 3. The PCR products were ligated to the *pCAMBIA1301* backbone vector using the In-Fusion cloning system (Takara, cat.no 638908). *pNRPD1::NRPD1-GFP* and *pNRPE1::NRPE1-GFP* constructs consisting 6.3 and 4.0 kb of promoter sequences, 5.9 and 8.4 kb of coding genomic sequences, 2.2 and 1.4 kb of downstream sequences, respectively, and sGFP sequences at C-terminus were generated in *pENTR2B* backbone (Invitrogen, cat.no: A10463) using subcloning and re-assemble strategy combining SLiCE reaction^68^ and restriction enzymes (Supplementary Data 3). Resultant constructs were subsequently cloned into a binary plant expression vector pGWB601^69^ via a Gateway LR reaction.

### AGO immunoprecipitation and sRNA preparation

Flower buds (up to flower stage 12), with open flowers carefully removed, from Arabidopsis plants transgenic for Venus-tagged AGO in either the wild-type or mutant backgrounds, were harvested on ice. Approximately 50 plants were frozen and ground into a fine powder using liquid nitrogen. The powder was then mixed with IP buffer (20 mM Tris-HCl (pH=7.5), 10 mM NP-40, 10% glycerol, 1 mM EDTA, 1 mM PMSF, 5 mM DTT and protease inhibitor (Roche, #11836170001)) and incubated on a rotating wheel for 30 min. This and all subsequent steps were performed at 4 °C. Cell debris was removed by centrifugation at 12,000 × *g* for 10 min, repeated twice. The supernatant was incubated with 50 μL GFP-trap magnetic beads (ChromoTek) on a rotating wheel for 2 h. Afterwards, the beads were washed 5 times with IP buffer. Finally, TRIzol reagent was used to elute immunoprecipitants and extract AGO-bound sRNAs, which were then prepared for library construction.

### Library preparation and high-throughput sequencing

AGO-immunoprecipitated sRNA libraries were prepared as previously reported^43^. All sRNA-seq libraries were constructed from two biological replicates. Bisulfite sequencing (BS-seq) libraries of meiocytes isolated from WT and different mutants were constructed as previously described^43^. RNA-seq libraries of WT, *ago5*, *ago4,6,9* and *pol-iv* meiocytes were prepared as previously reported^44^. All RNA-seq libraries were constructed from three biological replicates of isolated cells. All the above libraries were sequenced on the NextSeq 500 (Illumina) platform. Statistics of all libraries are listed in Supplementary Tables 1–3.

### Confocal microscopy

Anthers at stage 6 were dissected from floral buds in a drop of sterilized water under a stereo microscope (Zeiss Stemi 1000). Dissected anthers were examined using a Leica SP8 confocal microscope with 20× lens (HCX PL APO CS 20.0× 0.70 DRY UV; for AGO reporter lines) or a Zeiss LSM780 confocal microscope with a W-plan-Apochromat 40×/1.0 DIC objective (for NRPD/NRPE1 reporter lines). The following settings were used for the visualization of Venus (excitation at 514 nm, emission bandpass at 511–563 nm), autofluorescence (excitation at 514 nm, emission bandpass at 616–668 nm), GFP (excitation at 488 nm and emission bandpass at 488–559 nm), and RFP (excitation at 561 nm and emission bandpass at 567-646 nm).

### Meiotic phenotype analysis

Meiotic cell spreads were prepared using fixed flower buds as described previously^70^. Slides were stained with DAPI and analyzed using a Zeiss AxioScan slide scanning microscope. The formation of tetrads and triads was analyzed and quantified by two independent investigators in a double-blind manner.

### RT-PCR

300 ng total RNA was reverse transcribed with a RevertAid First Strand cDNA Synthesis Kit (Thermo Fisher Scientific, K1621) for RT–PCR (Supplementary Fig. 6a). RT–PCR was performed to detect the retention of the last intron in *MPS1* as previously described^44^.

### Data analysis

For BS-seq, adaptor, low-quality sequencing reads and the first 9 bp of each read were trimmed using TrimGalore version 0.4.1 with default parameters. Clean reads were then mapped to the TAIR10 Arabidopsis reference genome using Bismark^71^ (version 0.22.2). Duplicated reads were filtered out using Picard tools MarkDuplicates version 1.141 (http://broadinstitute.github.io/picard/). Subsequent DNA methylation analysis was performed as previously described. For sRNA-seq, adaptor trimming for raw sequencing reads was performed using Cutadapt^72^ (version 3.7). AGO-bound 18-28 nt sRNAs were mapped to the TAIR10 *Arabidopsis* reference genome with zero mismatches (-v 0) using ShortStack^73^ (V3.85). The abundance of 21-, 22- or 24-nt sRNAs loaded into each AGO at *pol-iv*-dependent clusters or different categories of *clsy*-dependent clusters (all defined in ref.^5^) was calculated using Reads per Kilobase Per Million (RPKM). The FastQC software (Version 0.12.0, https://www.bioinformatics.babraham.ac.uk/projects/fastqc/) was used for analysis of the 5′ terminal nucleotide composition for AGO-loaded sRNAs. For RNA-seq, reads were mapped to *Arabidopsis* reference transcriptome (TAIR10) using Hisat2^74^. FeatureCounts^75^ was used to calculate the number of raw counts for individual TEs or genes, and DEseq2^76^ was used to identify differentially expressed TEs or genes in the corresponding mutant versus WT. To minimize mapping artefacts from repetitive TE regions, TEs whose coverage was dominated by low-MAPQ multi-mappers and secondary alignments over poly(A/T) tracts were excluded.

### Statistical analysis

For all the violin plots in this study, the plot shows the distribution of the data and its probability density, with the box enclosing the middle 50% of the distribution (the horizontal line marks the median) and whiskers illustrating 1.5 times the interquartile range. Unless otherwise stated, *P* values in violin plots were all calculated using a two-sided Kolmogorov–Smirnov test. All reps included were biological replicates.

### Reporting summary

Further information on research design is available in the Nature Portfolio Reporting Summary linked to this article.

## Supplementary information

Supplementary information

Description of Additional Supplementary Files

Supplementary Data 1

Supplementary Data 2

Supplementary Data 3

Reporting Summary

Transparent Peer Review file

## Data Availability

Illumina sequencing data (RIP-seq, BS-seq, and RNA-seq) generated in this study have been deposited in the NCBI BioProject and are accessible under accession number PRJNA1192978. Published BS-seq data of Arabidopsis meiocyte used in this study for Fig. 4a are available in the NCBI GEO database under accession code GSE161625.

## Code availability

All custom codes are available via the request to the corresponding author.

## References

[1] Chen, X. & Rechavi, O. Plant and animal small RNA communications between cells and organisms. *Nat. Rev. Mol. Cell Biol.* **23**, 185–203 (2021). 10.1038/s41580-021-00425-y PMC9208737 34707241

[2] Zhan, J. & Meyers, B. C. Plant small RNAs: their biogenesis, regulatory roles, and functions. *Annu. Rev. Plant Biol.* **74**, 21–51 (2023). 10.1146/annurev-arplant-070122-035226 36854480

[3] Matzke, M. A. & Mosher, R. A. RNA-directed DNA methylation: an epigenetic pathway of increasing complexity. *Nat. Rev. Genet.* **15**, 394–408 (2014). 10.1038/nrg3683 24805120

[4] Felgines, L. et al. CLSY docking to Pol IV requires a conserved domain critical for small RNA biogenesis and transposon silencing. *Nat. Commun.* **15**, 10298 (2024). 10.1038/s41467-024-54268-0 PMC11603163 39604359

[5] Zhou, M., Palanca, A. M. S. & Law, J. A. Locus-specific control of the de novo DNA methylation pathway in Arabidopsis by the CLASSY family. *Nat. Genet.* **50**, 865–873 (2018). 10.1038/s41588-018-0115-y PMC6317521 29736015

[6] Martins, L. M. & Law, J. A. Moving targets: mechanisms regulating siRNA production and DNA methylation during plant development. *Curr. Opin. Plant Biol.* **75**, 102435 (2023). 10.1016/j.pbi.2023.102435 PMC10581331 37598540

[7] Zhou, M. et al. The CLASSY family controls tissue-specific DNA methylation patterns in Arabidopsis. *Nat. Commun.* **13**, 244 (2022). 10.1038/s41467-021-27690-x PMC8752594 35017514

[8] Fukudome, A. et al. Structure and RNA template requirements of Arabidopsis RNA-DEPENDENT RNA POLYMERASE 2. *Proc. Natl. Acad. Sci. USA* **118**, e2115899118 (2021). 10.1073/pnas.2115899118 PMC8713982 34903670

[9] Huang, K. et al. Pol IV and RDR2: a two-RNA-polymerase machine that produces double-stranded RNA. *Science* **374**, 1579–1586 (2021). 10.1126/science.abj9184 34941388

[10] Mishra, V. et al. Assembly of a dsRNA synthesizing complex: RNA-DEPENDENT RNA POLYMERASE 2 contacts the largest subunit of NUCLEAR RNA POLYMERASE IV. *Proc. Natl. Acad. Sci. USA* **118**, e2019276118 (2021). 10.1073/pnas.2019276118 PMC8020760 33753485

[11] Loffer, A. et al. A DCL3 dicing code within Pol IV-RDR2 transcripts diversifies the siRNA pool guiding RNA-directed DNA methylation. *eLife* **11**, e73260 (2022). 10.7554/eLife.73260 PMC8846587 35098919

[12] Wang, Q. et al. Mechanism of siRNA production by a plant Dicer-RNA complex in dicing-competent conformation. *Science* **374**, 1152–1157 (2021). 10.1126/science.abl4546 PMC8682726 34648373

[13] Liu, W. et al. RNA-directed DNA methylation involves co-transcriptional small-RNA-guided slicing of polymerase V transcripts in Arabidopsis. *Nat. Plants* **4**, 181–188 (2018). 10.1038/s41477-017-0100-y PMC5832601 29379150

[14] Sigman, M. J. et al. An siRNA-guided ARGONAUTE protein directs RNA polymerase V to initiate DNA methylation. *Nat. Plants* **7**, 1461–1474 (2021). 10.1038/s41477-021-01008-7 PMC8592841 34750500

[15] Zhang, H., Lang, Z. & Zhu, J. K. Dynamics and function of DNA methylation in plants. *Nat. Rev. Mol. Cell Biol.* **19**, 489–506 (2018). 10.1038/s41580-018-0016-z 29784956

[16] Li, Z. et al. Origin, evolution and diversification of plant ARGONAUTE proteins. *Plant J.* **109**, 1086–1097 (2022). 10.1111/tpj.15615 PMC9208301 34845788

[17] Wu, L. et al. Rice MicroRNA effector complexes and targets. *Plant Cell* **21**, 3421–3435 (2009). 10.1105/tpc.109.070938 PMC2798332 19903869

[18] Bohmert, K. et al. AGO1 defines a novel locus of Arabidopsis controlling leaf development. *EMBO J.* **17**, 170–180 (1998). 10.1093/emboj/17.1.170 PMC1170368 9427751

[19] Harvey, J. J. et al. An antiviral defense role of AGO2 in plants. *PLoS ONE* **6**, e14639 (2011). 10.1371/journal.pone.0014639 PMC3031535 21305057

[20] Zhang, Z., Liu, X., Guo, X., Wang, X. J. & Zhang, X. Arabidopsis AGO3 predominantly recruits 24-nt small RNAs to regulate epigenetic silencing. *Nat. Plants* **2**, 16049 (2016). 10.1038/nplants.2016.49 27243648

[21] Jullien, P. E. et al. Functional characterization of Arabidopsis ARGONAUTE 3 in reproductive tissues. *Plant J.* **103**, 1796–1809 (2020). 10.1111/tpj.14868 32506562

[22] Montgomery, T. A. et al. Specificity of ARGONAUTE7-miR390 Interaction and Dual Functionality in TAS3 Trans-Acting siRNA Formation. *Cell* **133**, 128–141 (2008). 10.1016/j.cell.2008.02.033 18342362

[23] Vaucheret, H. & Voinnet, O. The plant siRNA landscape. *Plant Cell* **36**, 246–275 (2024). 10.1093/plcell/koad253 PMC10827316 37772967

[24] Zhou, C. et al. The trans-acting short interfering RNA3 pathway and no apical meristem antagonistically regulate leaf margin development and lateral organ separation, as revealed by analysis of an argonaute7/lobed leaflet1 mutant in Medicago truncatula. *Plant Cell* **25**, 4845–4862 (2013). 10.1105/tpc.113.117788 PMC3903991 24368797

[25] Zhang, X. et al. Arabidopsis Argonaute 2 regulates innate immunity via miRNA393(*)-mediated silencing of a Golgi-localized SNARE gene, MEMB12. *Mol. Cell* **42**, 356–366 (2011). 10.1016/j.molcel.2011.04.010 PMC3101262 21549312

[26] Bradamante, G. et al. Two ARGONAUTE proteins loaded with transposon-derived small RNAs are associated with the reproductive cell lineage in Arabidopsis. *Plant Cell* **36**, 863–880 (2024). 10.1093/plcell/koad295 PMC10980394 38060984

[27] Jullien, P. E., Schroder, J. A., Bonnet, D. M. V., Pumplin, N. & Voinnet, O. Asymmetric expression of Argonautes in reproductive tissues. *Plant Physiol.* **188**, 38–43 (2022). 10.1093/plphys/kiab474 PMC8774725 34687292

[28] Liu, Y., Teng, C., Xia, R. & Meyers, B. C. PhasiRNAs in plants: their biogenesis, genic sources, and roles in stress responses, development, and reproduction. *Plant Cell* **32**, 3059–3080 (2020). 10.1105/tpc.20.00335 PMC7534485 32817252

[29] Pokhrel, S. et al. Pre-meiotic 21-nucleotide reproductive phasiRNAs emerged in seed plants and diversified in flowering plants. *Nat. Commun.* **12**, 4941 (2021). 10.1038/s41467-021-25128-y PMC8368212 34400639

[30] Song, X. et al. Roles of DCL4 and DCL3b in rice phased small RNA biogenesis. *Plant J.* **69**, 462–474 (2012). 10.1111/j.1365-313X.2011.04805.x 21973320

[31] Teng, C. et al. Dicer-like 5 deficiency confers temperature-sensitive male sterility in maize. *Nat. Commun.* **11**, 2912 (2020). 10.1038/s41467-020-16634-6 PMC7283321 32518237

[32] Zhai, J. et al. Spatiotemporally dynamic, cell-type-dependent premeiotic and meiotic phasiRNAs in maize anthers. *Proc. Natl. Acad. Sci. USA* **112**, 3146–3151 (2015). 10.1073/pnas.1418918112 PMC4364226 25713378

[33] Araki, S. et al. miR2118-dependent U-rich phasiRNA production in rice anther wall development. *Nat. Commun.* **11**, 3115 (2020). 10.1038/s41467-020-16637-3 PMC7305157 32561756

[34] Zhou, X. et al. 24-nt phasiRNAs move from tapetal to meiotic cells in maize anthers. *New Phytol.* **235**, 488–501 (2022). 10.1111/nph.18167 35451503

[35] Shi, C. et al. Temperature-sensitive male sterility in rice determined by the roles of AGO1d in reproductive phasiRNA biogenesis and function. *New Phytol.* **236**, 1529–1544 (2022). 10.1111/nph.18446 36031742

[36] Si, F. et al. Mobile ARGONAUTE 1d binds 22-nt miRNAs to generate phasiRNAs important for low-temperature male fertility in rice. *Sci. China Life Sci.* **66**, 197–208 (2023). 10.1007/s11427-022-2204-y 36239908

[37] Zhang, R. et al. CRISPR/Cas9-targeted mutagenesis of TaDCL4, TaDCL5 and TaRDR6 induces male sterility in common wheat. *Plant Biotechnol. J.* **21**, 839–853 (2023). 10.1111/pbi.14000 PMC10037139 36597709

[38] Zhang, Y. C. et al. Reproductive phasiRNAs regulate reprogramming of gene expression and meiotic progression in rice. *Nat. Commun.* **11**, 6031 (2020). 10.1038/s41467-020-19922-3 PMC7695705 33247135

[39] Jiang, P. et al. 21-nt phasiRNAs direct target mRNA cleavage in rice male germ cells. *Nat. Commun.* **11**, 5191 (2020). 10.1038/s41467-020-19034-y PMC7562718 33060587

[40] Komiya, R. et al. Rice germline-specific Argonaute MEL1 protein binds to phasiRNAs generated from more than 700 lincRNAs. *Plant J.* **78**, 385–397 (2014). 10.1111/tpj.12483 24635777

[41] Tamotsu, H., Koizumi, K., Briones, A. V. & Komiya, R. Spatial distribution of three ARGONAUTEs regulates the anther phasiRNA pathway. *Nat. Commun.* **14**, 3333 (2023). 10.1038/s41467-023-38881-z PMC10247740 37286636

[42] Xia, R. et al. 24-nt reproductive phasiRNAs are broadly present in angiosperms. *Nat. Commun.* **10**, 627 (2019). 10.1038/s41467-019-08543-0 PMC6367383 30733503

[43] Long, J. et al. Nurse cell-derived small RNAs define paternal epigenetic inheritance in Arabidopsis. *Science* **373**, eabh0556 (2021). 10.1126/science.abh0556 34210850

[44] Walker, J. et al. Sexual-lineage-specific DNA methylation regulates meiosis in Arabidopsis. *Nat. Genet.* **50**, 130–137 (2018). 10.1038/s41588-017-0008-5 PMC7611288 29255257

[45] Kawakatsu, T. et al. Unique cell-type-specific patterns of DNA methylation in the root meristem. *Nat. Plants* **2**, 16058 (2016). 10.1038/nplants.2016.58 PMC4855458 27243651

[46] Stroud, H. et al. Non-CG methylation patterns shape the epigenetic landscape in Arabidopsis. *Nat. Struct. Mol. Biol.* **21**, 64–72 (2014). 10.1038/nsmb.2735 PMC4103798 24336224

[47] Buttress, T. et al. Histone H2B.8 compacts flowering plant sperm through chromatin phase separation. *Nature* **611**, 614–622 (2022). 10.1038/s41586-022-05386-6 PMC9668745 36323776

[48] Sanders, P. M. et al. Anther developmental defects in Arabidopsis thaliana male-sterile mutants. *Sex. Plant Reprod.* **11**, 297–322 (1999).

[49] Wang, F. & Axtell, M. J. AGO4 is specifically required for heterochromatic siRNA accumulation at Pol V-dependent loci in Arabidopsis thaliana. *Plant J.* **90**, 37–47 (2017). 10.1111/tpj.13463 28002617

[50] Havecker, E. R. et al. The Arabidopsis RNA-directed DNA methylation argonautes functionally diverge based on their expression and interaction with target loci. *Plant Cell* **22**, 321–334 (2010). 10.1105/tpc.109.072199 PMC2845420 20173091

[51] Mi, S. et al. Sorting of small RNAs into Arabidopsis argonaute complexes is directed by the 5’ terminal nucleotide. *Cell* **133**, 116–127 (2008). 10.1016/j.cell.2008.02.034 PMC2981139 18342361

[52] Erdmann, R. M. & Picard, C. L. RNA-directed DNA methylation. *PLoS Genet.* **16**, e1009034 (2020). 10.1371/journal.pgen.1009034 PMC7544125 33031395

[53] Dubin, M. J. et al. DNA methylation in Arabidopsis has a genetic basis and shows evidence of local adaptation. *eLife* **4**, e05255 (2015). 10.7554/eLife.05255 PMC4413256 25939354

[54] Popova, O. V., Dinh, H. Q., Aufsatz, W. & Jonak, C. The RdDM pathway is required for basal heat tolerance in Arabidopsis. *Mol. Plant* **6**, 396–410 (2013). 10.1093/mp/sst023 PMC3603006 23376771

[55] Kawakatsu, T. et al. Epigenomic diversity in a global collection of Arabidopsis thaliana accessions. *Cell* **166**, 492–505 (2016). 10.1016/j.cell.2016.06.044 PMC5172462 27419873

[56] Naydenov, M. et al. High-temperature effect on genes engaged in DNA methylation and affected by DNA methylation in Arabidopsis. *Plant Physiol. Biochem.* **87**, 102–108 (2015). 10.1016/j.plaphy.2014.12.022 25576840

[57] Mari-Ordonez, A. et al. Reconstructing de novo silencing of an active plant retrotransposon. *Nat. Genet.* **45**, 1029–1039 (2013). 10.1038/ng.2703 23852169

[58] Cuerda-Gil, D. & Slotkin, R. K. Non-canonical RNA-directed DNA methylation. *Nat. Plants* **2**, 16163 (2016). 10.1038/nplants.2016.163 27808230

[59] Zhang, M. et al. CHH DNA methylation increases at 24-PHAS loci depend on 24-nt phased small interfering RNAs in maize meiotic anthers. *New Phytol.* **229**, 2984–2997 (2021). 10.1111/nph.17060 33135165

[60] Nonomura, K. et al. A germ cell specific gene of the ARGONAUTE family is essential for the progression of premeiotic mitosis and meiosis during sporogenesis in rice. *Plant Cell* **19**, 2583–2594 (2007). 10.1105/tpc.107.053199 PMC2002623 17675402

[61] Ono, S. et al. EAT1 transcription factor, a non-cell-autonomous regulator of pollen production, activates meiotic small RNA biogenesis in rice anther tapetum. *PLoS Genet.* **14**, e1007238 (2018). 10.1371/journal.pgen.1007238 PMC5825165 29432414

[62] Stroud, H., Greenberg, M. V. C., Feng, S., Bernatavichute, Y. V. & Jacobsen, S. E. Comprehensive analysis of silencing mutants reveals complex regulation of the Arabidopsis Methylome. *Cell* **152**, 352–364 (2013). 10.1016/j.cell.2012.10.054 PMC3597350 23313553

[63] Olmedo-Monfil, V. et al. Control of female gamete formation by a small RNA pathway in Arabidopsis. *Nature* **464**, 628–632 (2010). 10.1038/nature08828 PMC4613780 20208518

[64] Katiyar-Agarwal, S., Gao, S., Vivian-Smith, A. & Jin, H. A novel class of bacteria-induced small RNAs in Arabidopsis. *Genes Dev.* **21**, 3123–3134 (2007). 10.1101/gad.1595107 PMC2081978 18003861

[65] Onodera, Y. et al. Plant nuclear RNA polymerase IV mediates siRNA and DNA methylation-dependent heterochromatin formation. *Cell* **120**, 613–622 (2005). 10.1016/j.cell.2005.02.007 15766525

[66] Prusicki, M. A. et al. Live cell imaging of meiosis in Arabidopsis thaliana. *eLife* **8**, e42834 (2019). 10.7554/eLife.42834 PMC6559805 31107238

[67] Karimi, M., De Meyer, B. & Hilson, P. Modular cloning in plant cells. *Trends Plant Sci.* **10**, 103–105 (2005). 10.1016/j.tplants.2005.01.008 15749466

[68] Zhang, Y., Werling, U. & Edelmann, W. SLiCE: a novel bacterial cell extract-based DNA cloning method. *Nucleic Acids Res.* **40**, e55 (2012). 10.1093/nar/gkr1288 PMC3333860 22241772

[69] Nakamura, S. et al. Gateway binary vectors with the bialaphos resistance gene, bar, as a selection marker for plant transformation. *Biosci. Biotechnol. Biochem* **74**, 1315–1319 (2010). 10.1271/bbb.100184 20530878

[70] Armstrong, S. J., Sanchez-Moran, E. & Franklin, F. C. Cytological analysis of Arabidopsis thaliana meiotic chromosomes. *Methods Mol. Biol.* **558**, 131–145 (2009). 10.1007/978-1-60761-103-5_9 19685323

[71] Krueger, F. & Andrews, S. R. Bismark: a flexible aligner and methylation caller for Bisulfite-Seq applications. *Bioinformatics* **27**, 1571–1572 (2011). 10.1093/bioinformatics/btr167 PMC3102221 21493656

[72] Martin, M. Cutadapt removes adapter sequences from high-throughput sequencing reads. *EMBnet. J.* **17**, 10–12 (2011).

[73] Axtell, M. J. ShortStack: comprehensive annotation and quantification of small RNA genes. *RNA* **19**, 740–751 (2013). 10.1261/rna.035279.112 PMC3683909 23610128

[74] Kim, D., Langmead, B. & Salzberg, S. L. HISAT: a fast spliced aligner with low memory requirements. *Nat. Methods* **12**, 357–360 (2015). 10.1038/nmeth.3317 PMC4655817 25751142

[75] Liao, Y., Smyth, G. K. & Shi, W. featureCounts: an efficient general purpose program for assigning sequence reads to genomic features. *Bioinformatics* **30**, 923–930 (2014). 10.1093/bioinformatics/btt656 24227677

[76] Love, M. I., Huber, W. & Anders, S. Moderated estimation of fold change and dispersion for RNA-seq data with DESeq2. *Genome Biol.* **15**, 550 (2014). 10.1186/s13059-014-0550-8 PMC4302049 25516281

