# Supplementary material for: Multiple Argonaute complexes orchestrate epigenetic silencing in the Arabidopsis germline
Source: Nat Commun. 2026 Sep 12;17:10758. doi: 10.1038/s41467-026-77326-1 (PMC13661894; doi:10.1038/s41467-026-77326-1)
Supplement: Supplementary file 1 [file 41467_2026_77326_MOESM1_ESM.pdf]

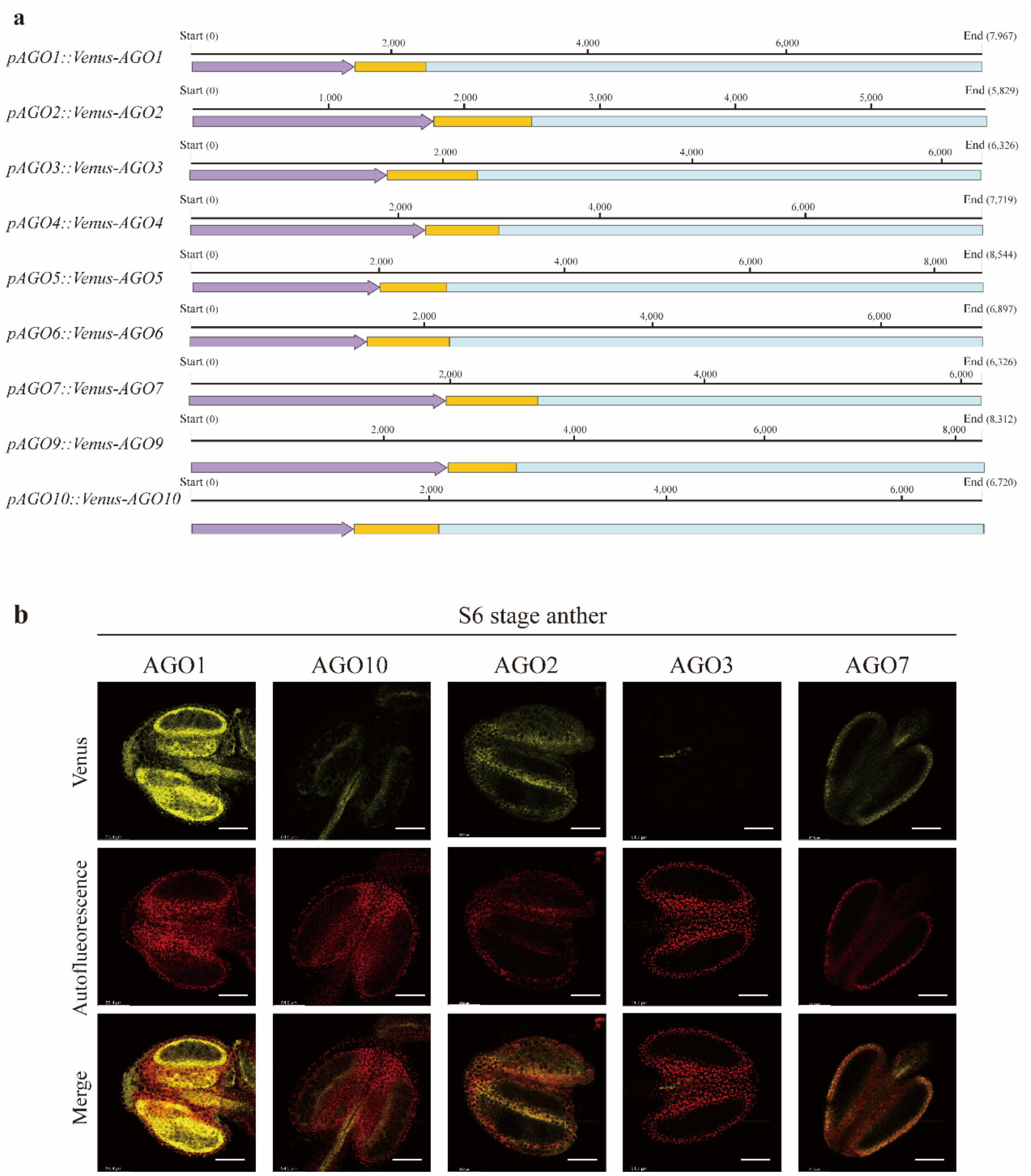

Supplementary Fig. 1 | AGO1/10/2/3/7 are barely detectable in meiocytes. a, DNA sequence used for making transgenic Venus-tagged AGO (AGO1-AGO10 excluding AGO8) reporter lines. **b,** Confocal images capturing Venus-AGO1/10/2/3/7 signals at Stage 6 anther of their corresponding reporter lines, scale bars=25μm.

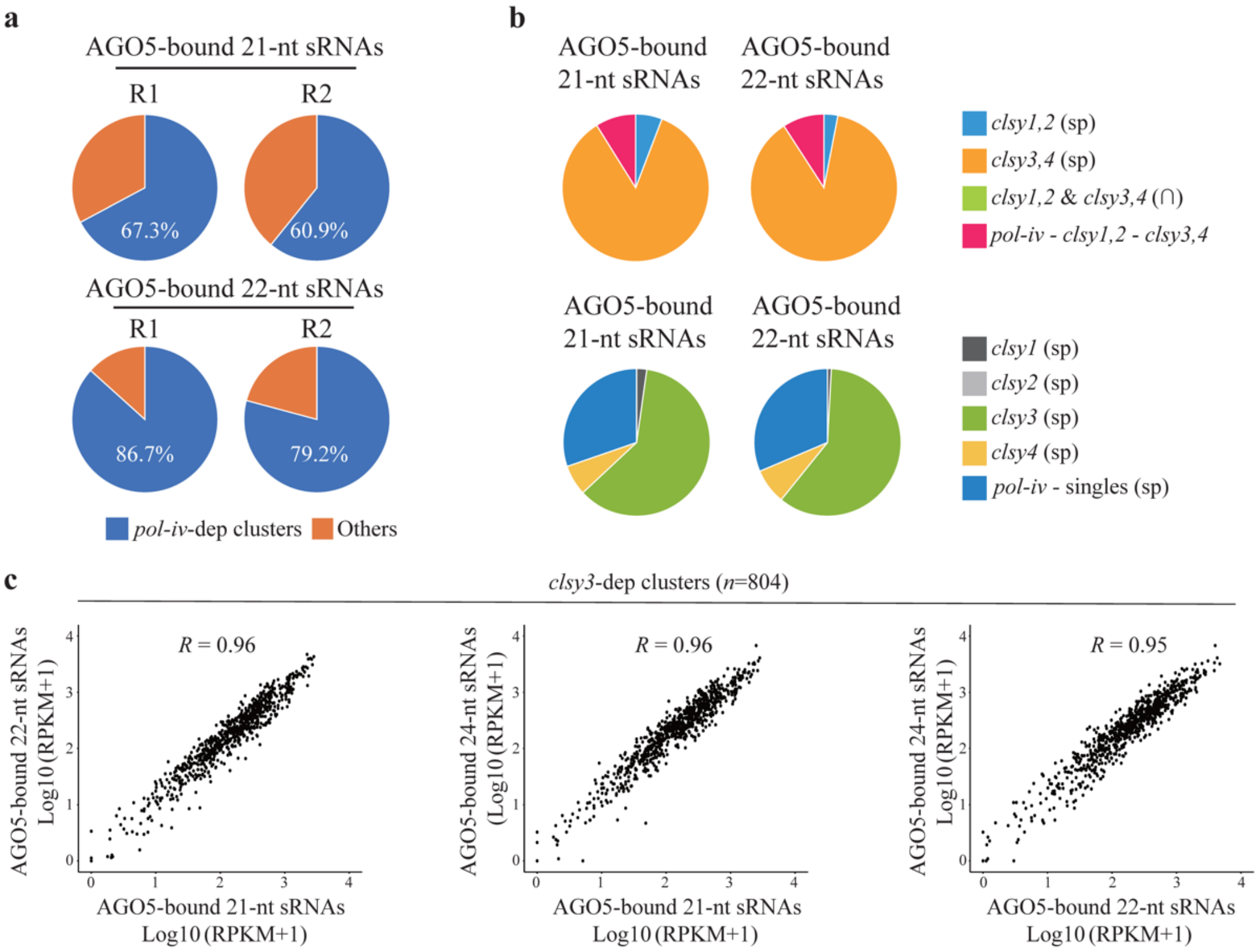

Supplementary Fig. 2 | The majority of AGO5-bound 21- and 22-nt sRNAs are derived from *pol-iv*-dependent sRNA clusters. a, Pie chart presenting the percentage of AGO5-bound 21- or 22-nt sRNAs that can be perfectly aligned to *pol-iv*-dep sRNA clusters accounting to total mapped AGO5-bound 21- or 22-nt sRNAs. The data of two biological replicates (R1 and R2) are shown. **b**, Pie chart showing the fraction of AGO5-bound 21- or 22-nt sRNAs that mapped to different classes of *clsy*-dep clusters accounting for those mapped to *pol-iv*-dep clusters. **c**, Scatter plots depicting correlations between abundance levels of AGO5-bound 21-, 22-, and 24-nt sRNAs abundance at *clsy3*-dep sRNA clusters. Pearson's *R* values are displayed.

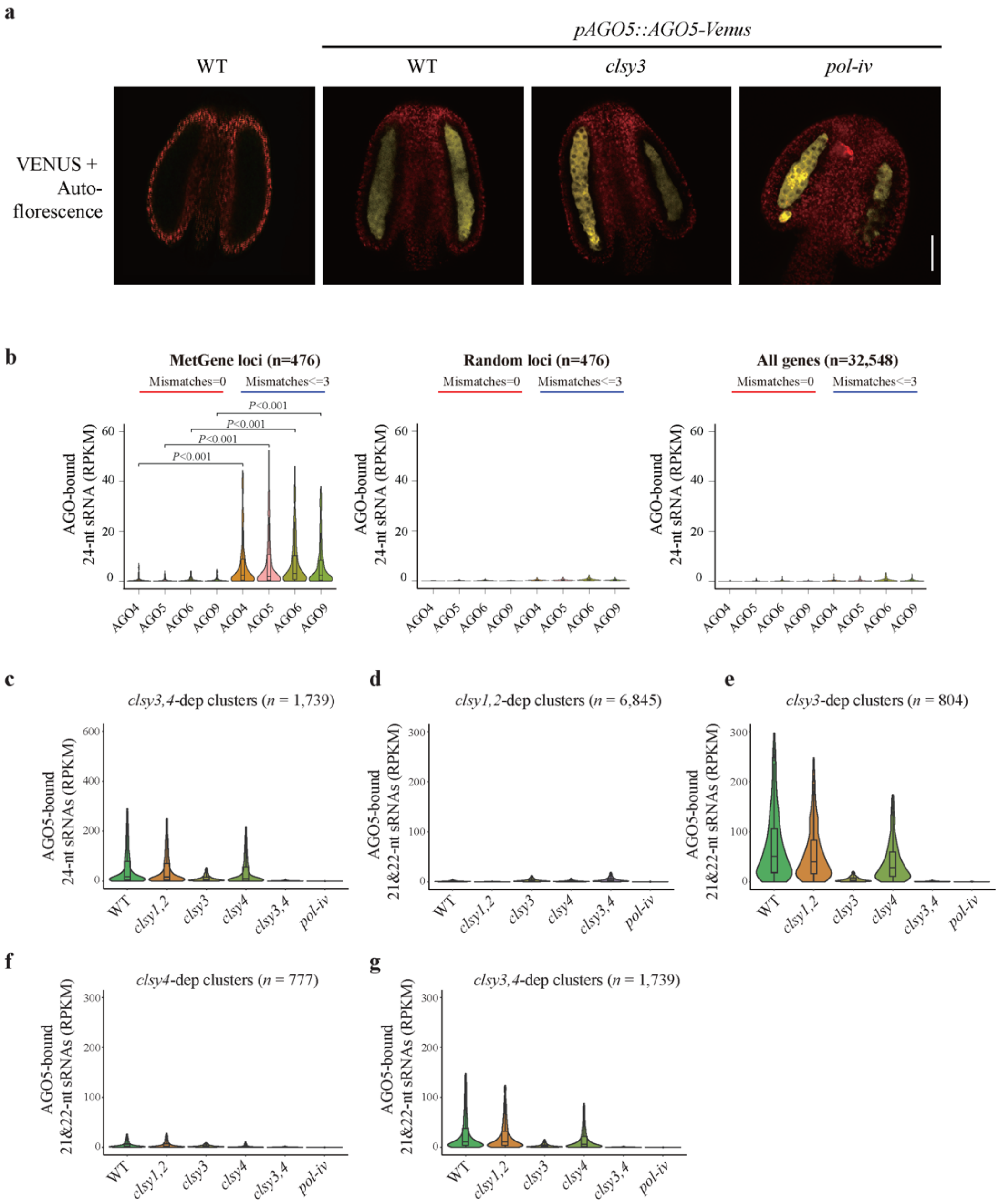

Supplementary Fig. 3 | Biogenesis of AGO5-bound siRNAs requires Pol IV and CLSY3. a, Confocal images of Stage 6 anthers in the indicated genotypes: WT, *AGO5-VENUS*, *AGO5-VENUS clsy3* and *AGO5-VENUS pol-iv* (from left to right). Scale bar, 25 µm. **b**, Boxplots showing the abundance of AGO-bound 24-nt sRNAs at MetGenes (left), a random set of loci matched for size and genomic features (middle), and all genes (right), with or without mismatches as indicated. **c**, Boxplots showing AGO5-bound 24-nt sRNA abundance at *clsy3,4*-dependent clusters in indicated genotypes. **d-g**, Boxplots showing the abundance of AGO5-bound 21&22-nt sRNAs at *clsy1,2*-dependent, *clsy3*-dependent, *clsy4*-dependent, and *clsy3,4*-dependent clusters. Box plots show the median (center line), interquartile range (box), and 1.5 × IQR whiskers. *P* values were calculated using a two-sided Kolmogorov–Smirnov test.

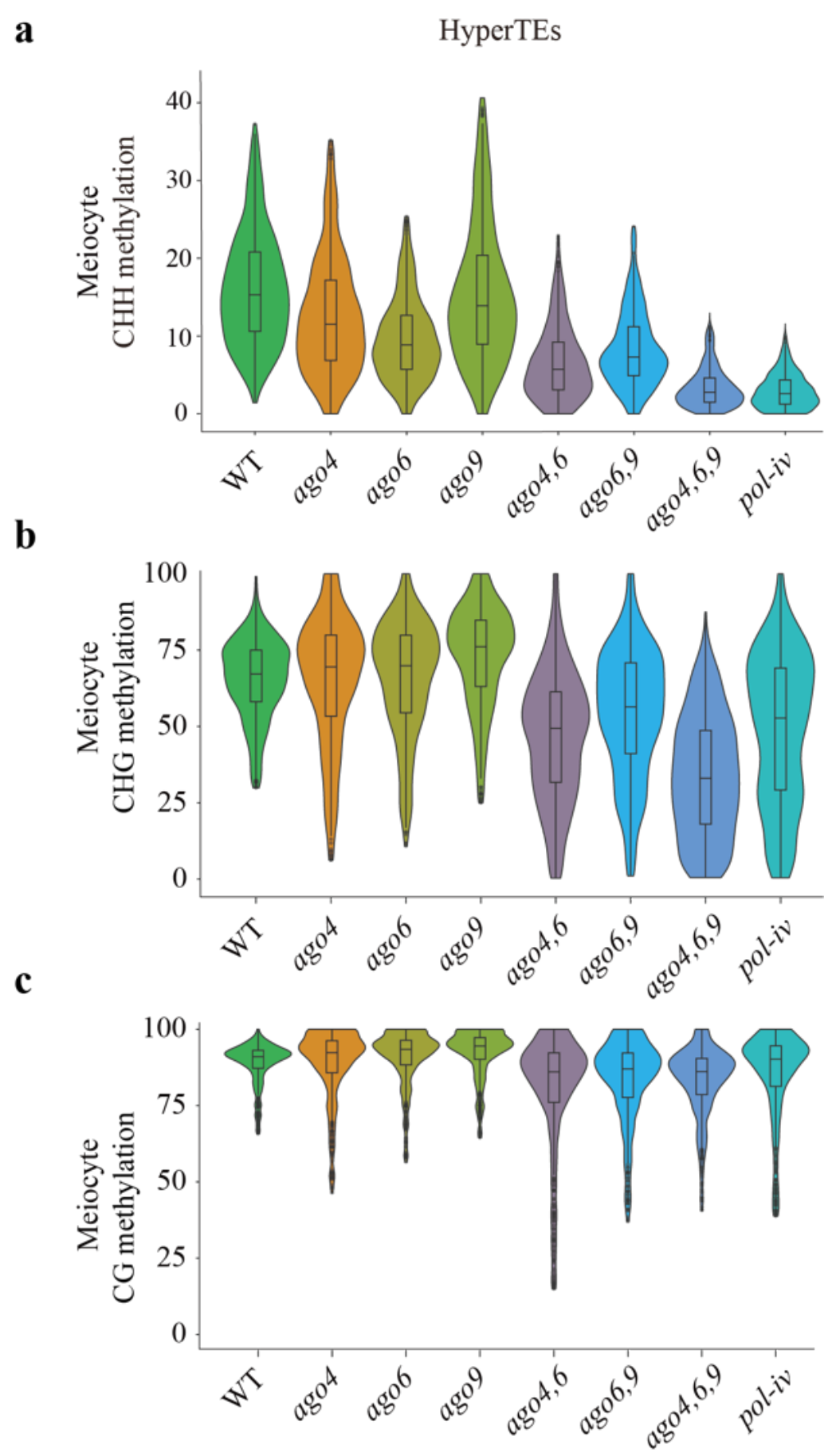

Supplementary Fig. 4 | AGO4/6/9 but not AGO5 are involved in RdDM in the meiocyte. a-c, Boxplots showing CHH (**a**), CHG (**b**) and CG (**c**) methylation levels at HyperTEs (n= 797) in male meiocytes from plants of indicated genotypes. Box plots show the median (center line), interquartile range (box), and 1.5 × IQR whiskers.

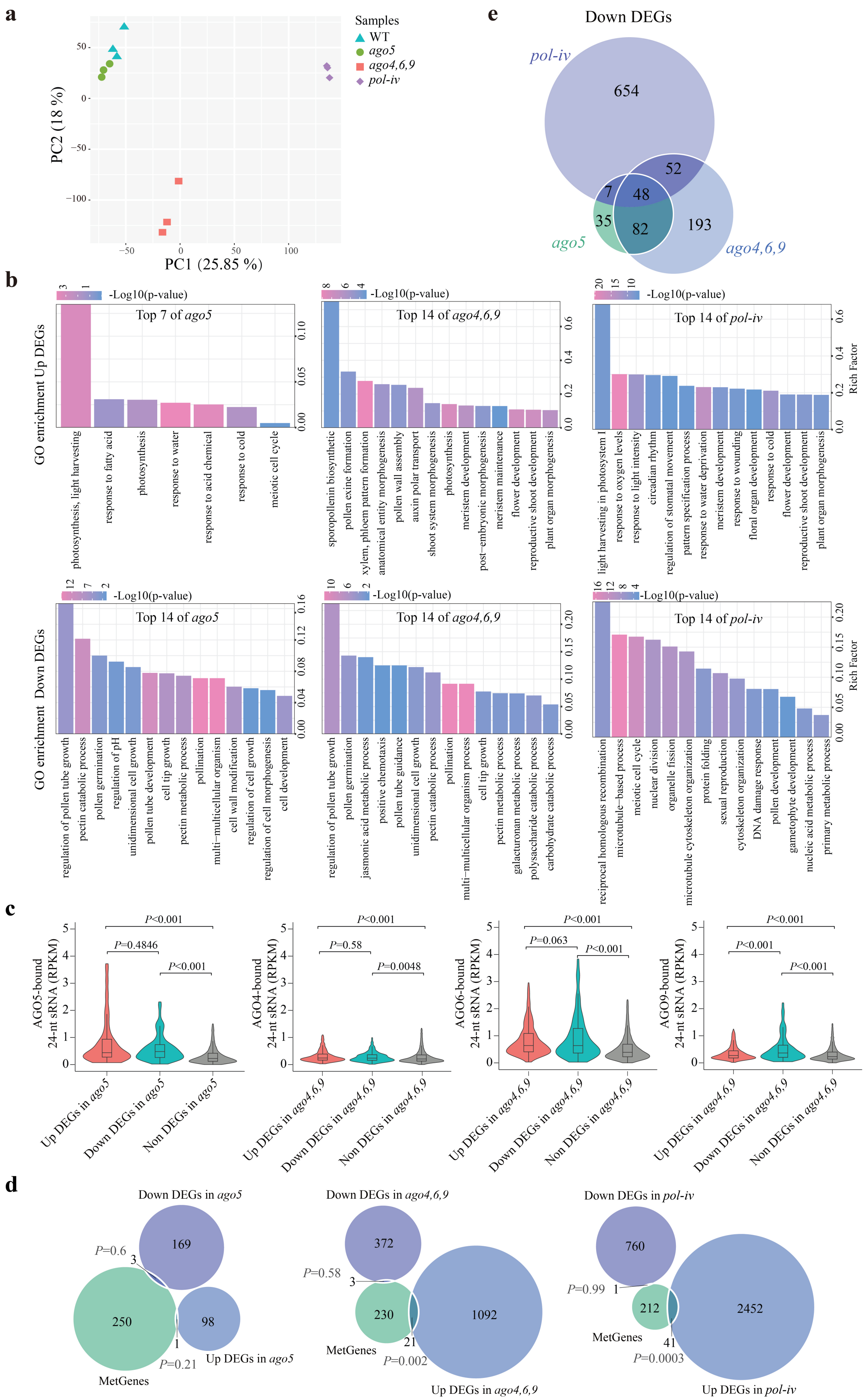
a
Samples
WT
ago5
ago4,6,9
pol-iv
PC1 (25.85 %)
PC2 (18 %)
e
Down DEGs
pol-iv
654
52
7
48
35
82
193
ago5
ago4,6,9
b
GO enrichment Up DEGs
-Log10(p-value)
Top 7 of ago5
Top 14 of ago4,6,9
Top 14 of pol-iv
GO enrichment Down DEGs
Top 14 of ago5
Top 14 of ago4,6,9
Top 14 of pol-iv
Rich Factor
c
AGO5-bound 24-nt sRNA (RPKM)
AGO4-bound 24-nt sRNA (RPKM)
AGO6-bound 24-nt sRNA (RPKM)
AGO9-bound 24-nt sRNA (RPKM)
Up DEGs in ago5
Down DEGs in ago5
Non DEGs in ago5
Up DEGs in ago4,6,9
Down DEGs in ago4,6,9
Non DEGs in ago4,6,9
d
Down DEGs in ago5
Up DEGs in ago5
MetGenes
Down DEGs in ago4,6,9
Up DEGs in ago4,6,9
Down DEGs in pol-iv
Up DEGs in pol-iv

35

Supplementary Fig. 5 | Differential gene expression analysis in WT, *ago5*, *ago4,6,9* and *pol-iv* meiocytes. **a**, PCA analysis of RNA-seq biological replicates from WT, *ago5*, *ago4,6,9* and *pol-iv* meiocytes. **b**, GO term analysis for upregulated or downregulated differentially expressed genes (DEGs) in *ago5*, *ago4,6,9* and *pol-iv* meiocytes compared to WT. *P* values were calculated using the hypergeometric test. **c**, Abundance of AGO-bound 24-nt sRNAs at DEGs that are upregulated in corresponding *ago* mutants compared to WT (Up DEGs), in comparison to genes that are downregulated (Down DEGs), or genes that are not differentially expressed (non-DEGs). Box plots show the median (center line), interquartile range (box), and 1.5 × IQR whiskers. *P* values were calculated using a two-sided Kolmogorov–Smirnov test. **d**, Venn diagram showing the overlap of MetGenes (a total of 254 genes overlap with MetGenes within their gene bodies) with DEGs identified in *ago5*, *ago4,6,9* and *pol-iv* meiocytes. *P* values were calculated using a hypergeometric test. **e**, Venn diagram showing the overlap of down-regulated genes identified in *ago5*, *ago4,6,9* and *pol-iv* meiocytes, compared to WT meiocytes.

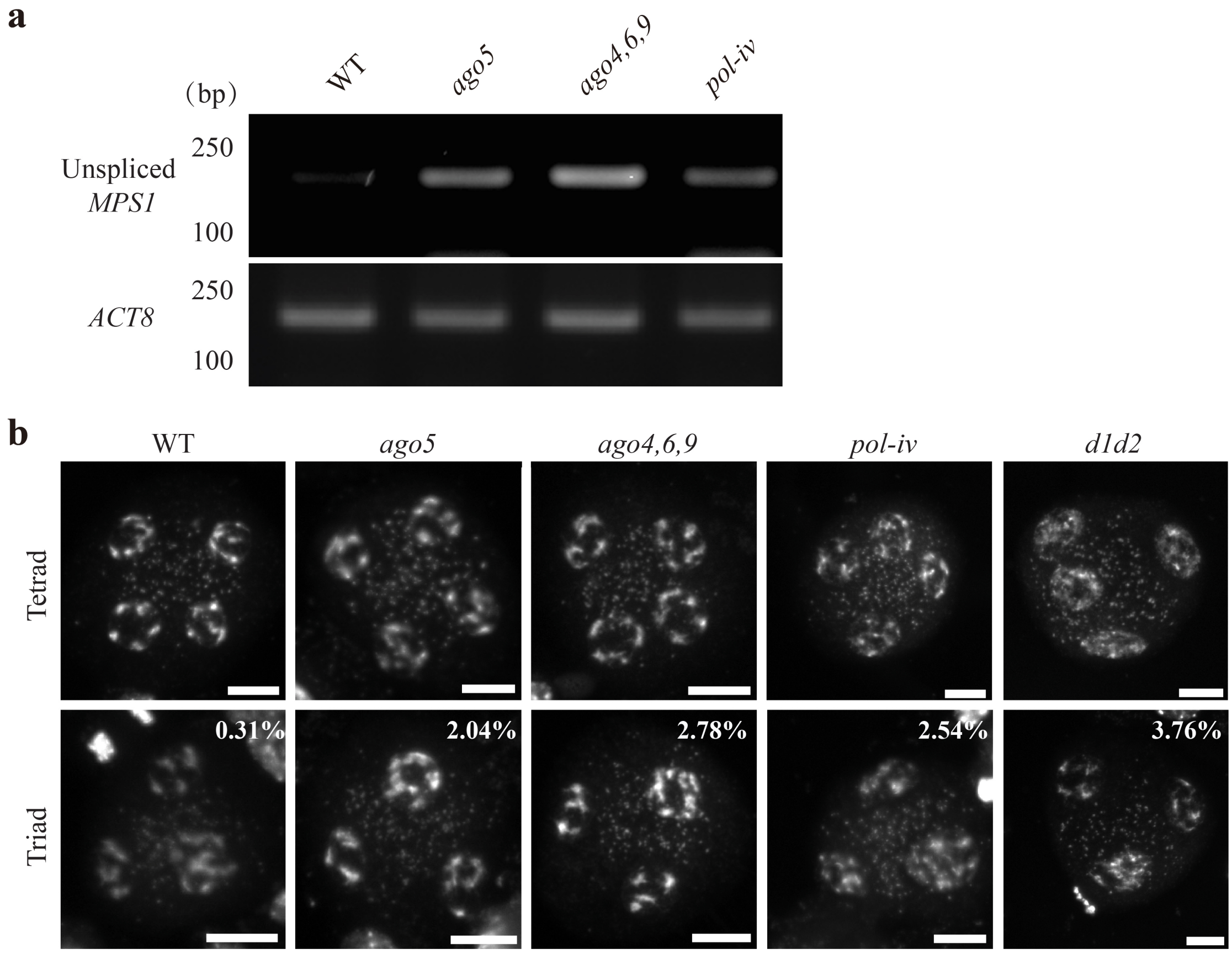

Supplementary Fig. 6 | *Ago5*, *ago4,6,9* and *pol-iv* mutant meiocytes exhibit increased *MPS1* mis-splicing and meiotic triad formation. a, RT-PCR of *MPS1* transcript with unspliced last intron in WT, *ago5*, *ago4,6,9* and *pol-iv* meiocytes. *ACTIN8* was used as a control. **b**, Representative DAPI-stained chromosome spreads from male meiocytes of indicated genotypes at telophase II. Percentages indicate the fraction of nuclei displaying triad phenotype. *d1d2*, *drm1 drm2* double mutant. Scale bars, 10 µm.

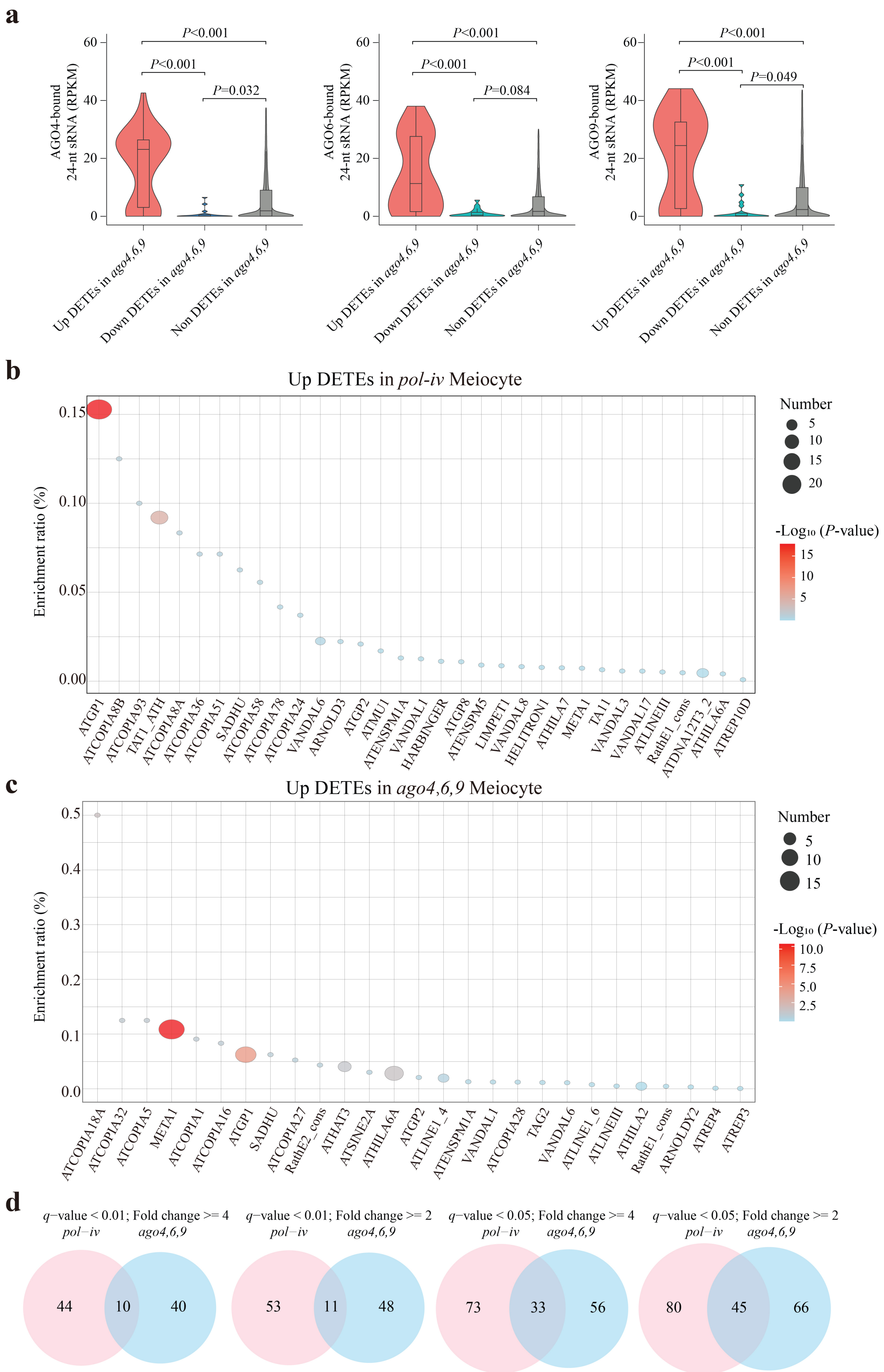

Supplementary Fig. 7 | TEs are up-regulated in *pol-iv* and *ago4,6,9* meiocytes. a, AGO4, 6, 9-bound siRNAs were significantly enriched at TEs that are upregulated in *ago4,6,9* triple mutant

compared to WT (Up DETEs), in comparison to TEs that are downregulated (Down DETEs), or TEs that are not differentially expressed (non-DETEs). Box plots show the median (center line), interquartile range (box), and 1.5 × IQR whiskers. *P* values were calculated using a two-sided Kolmogorov–Smirnov test. **b**,**c**, Enrichment analysis of TEs up-regulated in *pol-iv* (b) or *ago4,6,9* (c) meiocytes. *P* values of were calculated using the hypergeometric test. **d**, Corresponding figure for Fig. 6d, in which ~20% of upregulated TEs overlap between *ago4,6,9 and pol-iv* meiocytes under stringent cutoffs (*q*-value < 0.01 and Fold change >=4). This is likely an underestimate of overlap, as the overlap between upregulated TEs in *ago4,6,9* and *pol-iv* increases substantially when less stringent cutoffs are applied, indicating that many TEs are transcriptionally affected in both mutants but fall below the strict threshold in one condition.

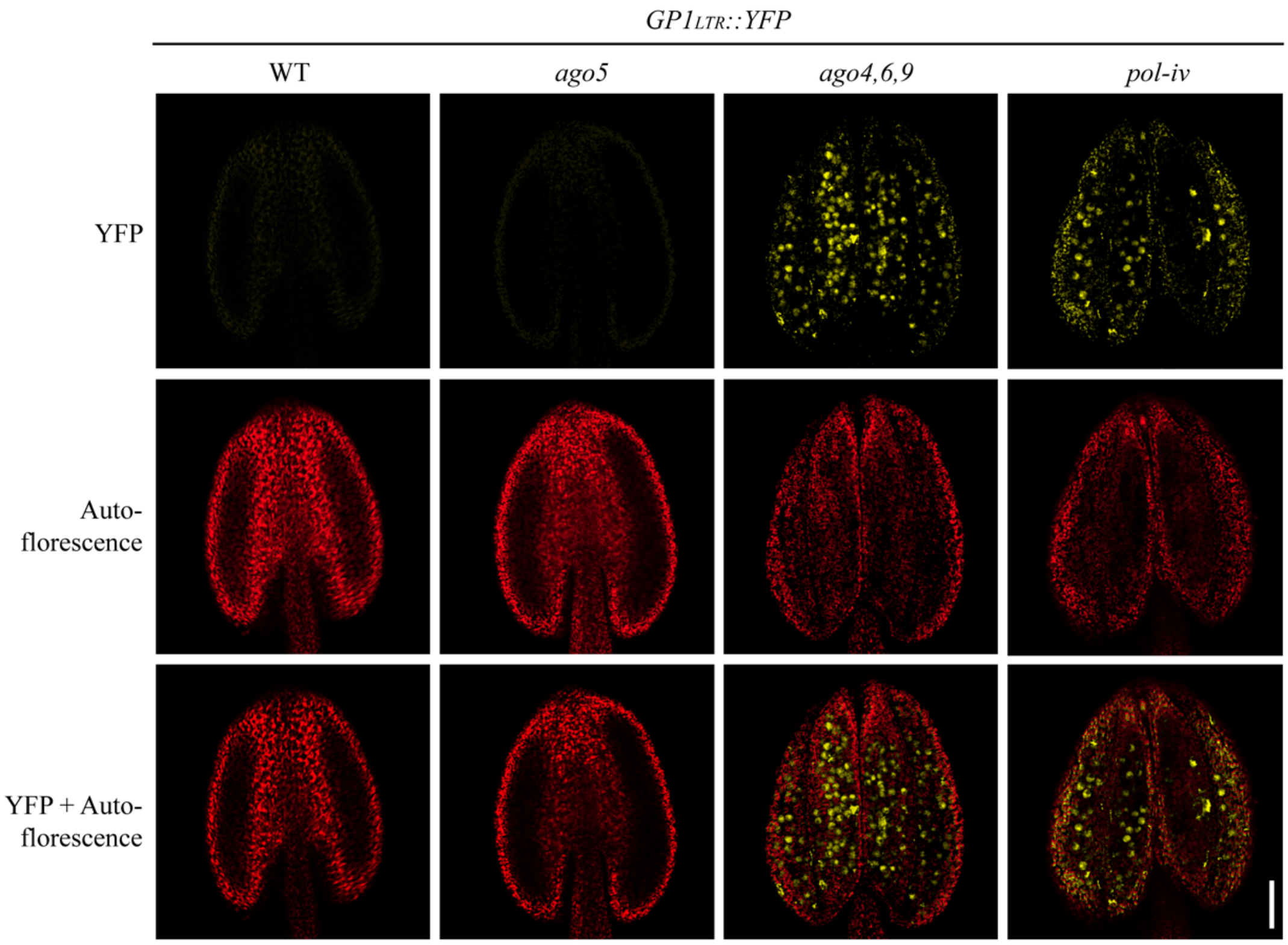

Supplementary Fig. 8 | AtGP1 AT2TE23670 is activated in *ago4,6,9* or *pol-iv* mutant germlines.
GP1$_{LTR}$::YFP anthers in indicated genetic backgrounds. Scale bars, 25 µm.

Supplementary Table 1. Summary statistics for RIP-seq libraries. Rep, biological replicate.

**Library Name**	**Raw reads**	**Clean reads (18-28 bp)**	**Mapped reads (18-28 bp)**	**Mapping rate (%)**
AGO4 RIP-seq in WT floral buds rep1	59,011,278	45,586,859	34,287,410	75.21%
AGO4 RIP-seq in WT floral buds rep2	41,503,321	33,518,837	27,033,281	80.65%
AGO5 RIP-seq in WT floral buds rep1	58,246,070	38,619,813	29,440,213	76.23%
AGO5 RIP-seq in WT floral buds rep2	63,118,823	37,707,966	29,136,806	77.27%
AGO6 RIP-seq in WT floral buds rep1	46,944,277	28,498,665	21,694,408	76.12%
AGO6 RIP-seq in WT floral buds rep2	100,820,542	50,277,153	39,086,108	77.74%
AGO9 RIP-seq in WT floral buds rep1	29,718,099	20,937,370	15,384,248	73.48%
AGO9 RIP-seq in WT floral buds rep2	32,813,249	23,997,113	18,018,179	75.08%
AGO5 RIP-seq in *clsy1,2* floral buds rep1	43,685,890	35,806,912	29,784,714	83.18%
AGO5 RIP-seq in *clsy1,2* floral buds rep2	36,469,968	14,877,343	10,221,449	68.70%
AGO5 RIP-seq in *clsy3* floral buds rep1	62,983,412	31,682,639	23,413,625	73.90%
AGO5 RIP-seq in *clsy3* floral buds rep2	55,240,730	37,025,411	31,476,199	85.01%
AGO5 RIP-seq in *clsy4* floral buds rep1	75,534,086	22,626,112	18,510,716	81.81%
AGO5 RIP-seq in *clsy4* floral buds rep2	72,304,817	27,467,470	21,552,151	78.46%
AGO5 RIP-seq in *clsy3,4* floral buds rep1	59,736,771	30,252,468	24,739,091	81.78%
AGO5 RIP-seq in *clsy3,4* floral buds rep2	57,031,825	29,979,911	25,090,623	83.69%
AGO5 RIP-seq in *pol-iv* floral buds rep1	53,268,034	18,643,132	13,420,919	71.99%
AGO5 RIP-seq in *pol-iv* floral buds rep2	61,804,087	18,369,545	12,697,589	69.12%
GFP RIP-seq in WT floral buds (*35S::GFP*) rep1	52,782,626	15,971,201	11,919,855	74.63%

GFP RIP-seq in WT floral buds (*35S::GFP*) rep2	43,389,964	14,164,358	11,386,174	80.39%

Supplementary Table 2. Summary statistics for BS-seq libraries. Rep, biological replicate.

**Library Name**	**Raw reads**	**Clean reads**	**Mapped reads**	**Mapping rate**	**Bisulfite conversion rate**
WT meiocytes BS-seq rep1	57,931,024	57,701,823	31,221,375	54.11%	99.58%
WT meiocytes BS-seq rep2	58,867,287	58,615,828	31,325,499	53.44%	99.73%
ago5 meiocytes BS-seq rep1	41,397,999	40,649,658	18,059,970	44.43%	99.64%
ago5 meiocytes BS-seq rep2	50,194,997	49,475,306	23,154,657	46.80%	99.60%
ago4 meiocytes BS-seq rep1	42,534,773	42,101,195	25,636,899	60.89%	99.35%
ago4 meiocytes BS-seq rep2	38,147,737	37,852,922	23,502,782	62.09%	99.45%
ago6 meiocytes BS-seq rep1	48,928,251	48,427,864	30,097,448	62.15%	99.37%
ago6 meiocytes BS-seq rep2	40,751,443	40,483,622	26,365,337	65.13%	99.46%
ago9 meiocytes BS-seq rep1	43,812,367	43,566,306	27,658,867	63.49%	99.46%
ago9 meiocytes BS-seq rep2	44,254,353	43,941,929	28,099,158	63.95%	99.31%
ago6,9 meiocytes BS-seq rep1	42,923,669	42,106,321	22,299,149	52.96%	99.61%
ago6,9 meiocytes BS-seq rep2	49,835,290	48,831,190	26,185,972	53.63%	99.62%
ago4,6 meiocytes BS-seq rep1	39,062,413	38,084,225	19,626,031	51.53%	99.64%
ago4,6 meiocytes BS-seq rep2	30,827,994	30,313,739	15,346,540	50.63%	99.64%
ago4,6,9 meiocytes BS-seq rep1	66,399,208	64,955,444	28,024,827	43.14%	99.64%

ago4,6,9 meiocytes BS-seq rep2	77,457,290	75,799,732	41,477,773	54.72%	99.64%
pol-iv meiocytes BS-seq rep1	44,045,004	43,819,143	25,033,362	57.13%	99.37%
pol-iv meiocytes BS-seq rep2	45,033,341	44,579,463	25,245,943	56.63%	99.60%

Supplementary Table 3. Summary statistics for RNA-seq libraries. Rep, biological replicate.

Library Name	Raw reads	Clean Reads	Mapped Reads	Mapping Rate
WT meiocytes RNA-seq rep1	29,755,163	29,598,139	28,605,948	96.65%
WT meiocytes RNA-seq rep2	24,844,194	24,696,089	24,005,137	97.20%
WT meiocytes RNA-seq rep3	21,954,776	21,864,576	21,102,823	96.52%
ago5 meiocytes RNA-seq rep1	23,106,089	23,024,285	22,368,946	97.15%
ago5 meiocytes RNA-seq rep2	22,333,369	22,274,726	21,626,094	97.09%
ago5 meiocytes RNA-seq rep3	23,501,503	23,359,258	22,531,606	96.46%
ago4,6,9 meiocytes RNA-seq rep1	33,702,304	32,683,946	31,688,993	96.96%
ago4,6,9 meiocytes RNA-seq rep2	29,225,006	28,546,665	27,644,339	96.84%
ago4,6,9 meiocytes RNA-seq rep3	17,113,990	17,089,362	16,609,479	97.19%
pol-iv meiocytes RNA-seq rep1	21,828,197	21,651,317	19,906,936	91.94%
pol-iv meiocytes RNA-seq rep2	21,092,719	21,016,104	19,202,379	91.37%
pol-iv meiocytes RNA-seq rep3	18,900,343	18,829,446	17,115,616	90.90%

Supplementary Table 4. Frequency of meiotic triads in indicated genotypes. *P* value, Fischer's exact test. Significant P values ($P < 0.05$) are indicated by asterisks.

	Triads (%)	n= total observations	*P* value vs WT	*P* value vs *drm1drm2*
ago5	2.04%	393	0.046*	0.225
ago4,6,9	2.78%	396	0.015*	0.504
pol-iv	2.54%	276	0.027*	0.467
drm1drm2	3.76%	266	0.003*	
WT	0.31%	324		0.003*
