# Supplementary material for: Multiple Argonaute complexes orchestrate epigenetic silencing in the Arabidopsis germline
Source: Nat Commun. 2026 Sep 12;17:10758. doi: 10.1038/s41467-026-77326-1 (PMC13661894; doi:10.1038/s41467-026-77326-1)
Supplement: Supplementary file 2 [file 41467_2026_77326_MOESM2_ESM.pdf]

Description of Additional Supplementary Files:

Title: Supplementary Data 1.

Description: Differentially expressed genes in *pol-iv, ago4,6,9* triple and *ago5* mutants compared to WT.

Title: Supplementary Data 2.

Description: Differentially expressed transposons in *pol-iv*, *ago4,6,9* triple and *ago5* mutants compared to WT.

Title: Supplementary Data 3.

Description: Primers used in this study.
