# Supplementary material for: Multiple Argonaute complexes orchestrate epigenetic silencing in the Arabidopsis germline
Source: Nat Commun. 2026 Sep 12;17:10758. doi: 10.1038/s41467-026-77326-1 (PMC13661894; doi:10.1038/s41467-026-77326-1)
Supplement: Supplementary file 4 [file 41467_2026_77326_MOESM7_ESM.pdf]

nature portfolio

Peer Review File

Multiple Argonaute complexes orchestrate epigenetic silencing in the Arabidopsis germline

Corresponding Author: Professor Xiaoqi Feng

This file contains all reviewer reports in order by version, followed by all author rebuttals in order by version.

Version 0:

Reviewer comments:

Reviewer #1

(Remarks to the Author)
Long et al. investigate the roles of AGO4, AGO5, AGO6, and AGO9 in regulating DNA methylation and gene expression in meiocytes using RIP-seq, WGBS-seq, and RNA-seq. While the study provides robust descriptive data, its novelty is limited by similarities to prior reports in somatic/gametic tissues. Critically, the authors fail to mechanistically explain how these AGOs collaborate during meiosis or resolve contradictions with recent findings (e.g., AGO5-sRNA associations in sperm cells). The lack of ago4/5/6/9 mutant analysis further weakens claims of synergy.

Major concerns:
1. 1. According to the Methods section (page 18), all RIP-seq data were derived from flower buds with only open flowers removed. Notably, AGO4, AGO6, AGO9, and AGO5 are also expressed in later-developed pollen and female gametophytes. More importantly, RIP-seq data of AGO5 and AGO9 in sperm cells reported by Herridge et al. (2025, NSMB) show that AGO5 primarily binds 20–22 nt sRNAs, which contradicts the results here (Fig. 2a). This raises questions about whether the RIP-seq profiles truly represent AGO5 in meiocytes, as the sRNA pool bound by AGO5 might be contaminated by signals from other cell types.
2. In P7 and the discussion section, the authors claim twice that "the increased dosage of Pol V pathway components could contribute to the pronounced off-target methylation effects." While Fig. 1c shows that Pol V and AGO4/6/9 are indeed enriched in meiocytes, there is no evidence demonstrating that overexpression of Pol V or AGO4/6/9 increases off-target methylation effects in somatic cells.
3. In Figure 3a, do Pol IV and CLSY3 affect AGO5 protein abundance?
4. The results in Fig. 4 demonstrate functional redundancy but not a "dosage-dependent role" of AGO4, AGO6, and AGO9.
5. In Fig. 6d, the upregulated TEs in ago4,6,9 do not overlap well with those in pol-iv, whereas the DEGs (Fig. 5a–d) show significant overlap between these two mutants. How can this result be explained? Additionally, the methylation status of those upregulated TEs (Fig. 6g) does not show significant loss.
6. In the ago5 mutant, what might explain the higher number of downregulated genes compared to upregulated genes? Furthermore, the only upregulated TE in the ago5 mutant is AT5TE50260, which belongs to LTR/Copia. Does this TE belong to the HyperTE loci?
7. Most importantly, do ago5, *ago4/6/9*, and pol IV mutants exhibit any defects in meiosis and subsequent male germ cell development? The corresponding author's lab has previously reported meiotic abnormalities in RdDM mutants in their Nature Genetics (2018) paper.

Reviewer #2

(Remarks to the Author)
In this manuscript, Long et al investigate the functions and biogenesis of siRNAs and argonautes in Arabidopsis male meiocytes (cells undergoing meiosis). They first identify the four most strongly expressed Ago genes in meiocytes and then carry out RIP-seq for each of the four to characterize their associated siRNAs. They focus on one, Ago5, that behaves differently than the others, and they carry out RIP-seq on Ago5 in multiple different mutant backgrounds including double mutants. This is a major accomplishment. They also carry out RNA-seq in the in an Ago triple mutant and in the single Ago5 mutant to investigate effects on both gene and TE expression. The major conclusions of the study focus on siRNAs that were previously reported to be transcribed by pol IV in the surrounding tapetal cells and transferred into meiocytes where

they direct gene silencing even with up to three mismatches between siRNAs and targets. Long et al call the target genes "MetGenes". They propose an interesting model for why silencing with mismatches could be tolerated in meiocytes but not in other cells: spatial separation of siRNA synthesis from siRNA targeting prevents amplification of the silencing signal, which would otherwise cause an off-target silencing problem or a heritable silencing problem for mismatched siRNA targeting. The work is extensive and detailed and appears to have been done rigorously.

I have one major concern regarding lack of direct evidence to connect specific siRNAs and Agos with differential gene expression in mutants. While previous work has already reported that pol IV-derived siRNAs can target MetGenes, and Long et al demonstrate that Ago5 binds pol IV-derived siRNAs, they do not determine whether Ago5 siRNAs have the potential to target MetGenes. An analysis should be included that shows the proportion of siRNAs from each Ago that have the potential to target MetGenes (allowing up to three mismatches), and the distributions of targeting-siRNAs per gene. Similarly, if differentially expressed (DE) genes that Long et al have identified in this manuscript are directly regulated by ago4,6,9 or ago5, the siRNAs from these argonautes should match the DE genes, independently of whether they qualify as MetGenes. This should be tested by plotting the distribution of siRNA abundance for DE genes from each ago RIP-seq dataset. This should be done separately for downregulated, upregulated, and non-DE genes. Paralell analyses should be done for TEs as well. This will allow a better way to evaluate the potential significance of Ago5 in terms of gene regulation, as well as the other three Agos.

A moderate concern is that in many places the text seems overly qualitative. In places where I would expect numbers there are just descriptive terms. Could words like "preferentially", "a subset", "a fraction", "largely", "most", and "slight" be supplemented with numbers that define them? Or is the source data not accurately quantifiable?

Of lesser concern;

The title is not particularly informative. Don't Multiple Argonaute complexes orchestrate epigenetic silencing in any tissue?

A bit more background on MetGenes is needed to understand this paper. How many have been identified? How many are differentially expressed in siRNA mutants and methylation mutants in meiocytes? What parts of the genes are differentially methylated between tapetum and meiocytes? Coding DNA? Introns? Promoters? Is the differential methylation in all three major contexts (CHH, CHG, and CG)?

Is the published RNA-seq meiocyte data from the same stage as meiocytes prepared for this study? What stage is that?

Specify whether Venus Ago fusion genes were encoded with full-length Ago genomic DNA including introns or just CDS only.

Figure 1: Error bar sizes? Define RFP-TAU5. Panel D is a little confusing becuase it makes it look like the siRNAs that target MetGenes are secondary siRNAs derived from HyperTEs in the meiocyte rather than siRNAs transferred from the tapetum.

Figure 2: The categorization schemes and labels in panels C and D are hard to understand. Maybe more detail in the legend would fix this, but it may require rethinking the figure. Similar problem in Figure 3D.

It is also hard to understand the Y axis scale in Figure 3B. What are the values normalized by? The legend indicates the values are "relative to the total number derived from pol-iv- dependent clusters". But if this is true, the total sum of the values for pol-iv-dependent clusters should be constant across genotypes, including in the pol iv mutant itself. What the plots in fact show is near-zero values for pol-iv-dependent clusters for the pol iv mutant, which suggests something else is being used as the normalizing factor.

In Figure 4, the number of loci included in each distribution should be indicated. Also it's not clear what the top colored segments in panel B are showing.'

While 50 TEs are upregulated in ago4,6,9 vs. WT, 21 TEs are also downregulated. Since siRNAs are predicted to suppress gene expression, this suggests that 2/5ths of the differential expression in this triple mutant is indirect. It would be worth mentioning this caveat in the interpretation. Also related to interpretation, even if one generously includes all 104 upregulated TEs in the number, does that support the statement "AGO4/6/9 and Pol IV are key regulators of TE silencing in meiocytes"? If the vast majority of TEs are not activated in the mutants, is it fair to call the Agos "key regulators"? Could one conclude the opposite, that they are largely; dispensable regulators in meiocytes?

Spatial separation of siRNAs from their Ago proteins would make them susceptible to degradation by nucleases. Do Long et al have any ideas on how the siRNAs avoid degradation in tapetal cells before transfer to the meiocytes for loading onto Agos?

Does the Ago triple mutant, the Ago5 single mutant, or the pol IV single mutant have a male fertility defect? Either way, this should be mentioned in the text.

Version 1:

Reviewer comments:

Reviewer #1

(Remarks to the Author)
In this revision, the authors have included additional experimental results, particularly refined AGO5 RIP-seq comparisons, new evidence supporting Pol IV–dependent DNA methylation, Venus-AGO5 imaging in pol IV and clsy3 backgrounds, reporter-based validation, and improved data analysis. Moreover, they have added explanations to clarify several previously confusing observations. These additions strengthen the study, and my previous concerns have been largely addressed. I therefore recommend the manuscript for publication, with the following points to be addressed:

Considering the very weak meiosis phenotype in the corresponding mutants, please provide a brief GO analysis (indicating which specific meiotic processes the deregulated genes are involved in) for the genes identified as deregulated (e.g., MetGene) in these mutants. The biological significance of these deregulated genes should also be discussed.

Please ensure that all figure legends involving statistical plots (e.g., violin plots, box plots) consistently report statistical methods, sample sizes, and p-value calculations throughout the manuscript. For example, the figure legend for Extended Data Fig. 3b should include the statistical test used, a description of how p-values were calculated, and a clear indication of what the box plot elements represent (e.g., median, quartiles).

Briefly clarify in the Results or Discussion section why different statistical thresholds lead to substantial differences in transposable element (TE) overlap, to help guide interpretation and avoid potential confusion.

Reviewer #2

(Remarks to the Author)
Long et al have mostly addressed my previous concerns in their rebuttal. I am surprised though that some of the data they put into the rebuttal they did not also put into or mention in the revised manuscript. In particular, ago mutant DE genes, unlike DE TEs, have similar numbers of Ago-bound siRNAs regardless of whether they are up or down regulated in mutants, and there are relatively small differences in numbers of Ago-bound siRNAs between DE genes and non-DE genes. It seems important that those numbers be included for the reader to consider. Not necessary but helpful would be some discussion too. Why would so many genes be targeted by Ago-bound siRNAs but down-regulated or not affected in ago mutants? It would also strengthen the manuscript to explicitly describe the amount of overlap between MetGenes and each group of DEGs, both up and downregulated ones. The manuscript clearly shows enrichment in overlaps between the different mutant DEGs, but leaves out the enrichment between DEGs and MetGenes. This, along with the above results, would help clarify how relevant the mutant DE analyses are to understanding the MetGene siRNA pathway.

Regarding this statement: "genes located near MetGenes loci (<1.2 kb) exhibited a clear trend of transcriptional up-regulation in pol-iv and ago4,6,9 meiocytes, but not in ago5 meiocytes (Fig. 5g)": Are MetGenes and MetGene loci the same thing? Since there are only 99 Ago5-upregulated DEGs, I would not expect to see an ago5 mutant effect on the entire MetGene set in this kind of metagene analysis. The signal of those 99 genes would be too subtle relative to the ~1000 genes upregulated in the ago triple mutant. Related to this, the significance of Fig. 5g is hard to evaluate. Is it supposed to demonstrate that Ago5 does not impact expression of flanking genes, but Ago4,6,9 do? If so, the analysis should be repeated with just the 99 Ago5-upregulated DEGs instead of all MetGenes. The authors should also consider the possibility that a lot of MetGenes are poorly annotated and their TSSs and polyadenylation sites are incorrect, and they should consider the possibility that some upregulated MetGenes or DEGs are actually misannotated HyperTEs (TEs that are targeted by the same siRNA pathway in meiocytes).

Fig. 3a: Tracks 1 and 3 (WT R1 and clsy1,2 R1) look oddly similar. Is this an error in data analysis, or are these samples indeed so similar?

About this statement "the ago5 mutant also displayed a modest but significant increase in triad formation"--is it so modest that it cannot be quantified like ago4,6,9 triple mutant and pol-iv mutants? Both of those are reported in the text to have 4% triad formation.

Version 2:

Reviewer comments:

Reviewer #1

(Remarks to the Author)
I have no further concerns.

Reviewer #2

(Remarks to the Author)
I have no further comments.

Open Access This Peer Review File is licensed under a Creative Commons Attribution 4.0 International License, which permits use, sharing, adaptation, distribution and reproduction in any medium or format, as long as you give appropriate credit to the original author(s) and the source, provide a link to the Creative Commons license, and indicate if changes were made.
In cases where reviewers are anonymous, credit should be given to 'Anonymous Referee' and the source.
The images or other third party material in this Peer Review File are included in the article's Creative Commons license, unless indicated otherwise in a credit line to the material. If material is not included in the article's Creative Commons license and your intended use is not permitted by statutory regulation or exceeds the permitted use, you will need to obtain permission directly from the copyright holder.
To view a copy of this license, visit https://creativecommons.org/licenses/by/4.0/

We thank the reviewers for their constructive comments. Based on these, we have performed additional experiments and analyses, which have significantly improved the manuscript. Below we reproduce each of the reviewers' comments in italics, followed by our response.

REVIEWER COMMENTS

Reviewer #1 (Remarks to the Author):

Long et al. investigate the roles of AGO4, AGO5, AGO6, and AGO9 in regulating DNA methylation and gene expression in meiocytes using RIP-seq, WGBS-seq, and RNA-seq. While the study provides robust descriptive data, its novelty is limited by similarities to prior reports in somatic/gametic tissues. Critically, the authors fail to mechanistically explain how these AGOs collaborate during meiosis or resolve contradictions with recent findings (e.g., AGO5-sRNA associations in sperm cells). The lack of ago4/5/6/9 mutant analysis further weakens claims of synergy.

We thank the reviewer for their careful evaluation of our study and for acknowledging the robustness of our datasets. We respectfully disagree, however, with the notion of limited novelty due to similarities to prior reports. To our knowledge, our manuscript reports several key findings that have not been previously discovered: we demonstrate that 1) male meiotic siRNAs are concurrently associated with multiple Argonaute proteins that are highly enriched in meiocytes, including AGO4, AGO5, AGO6 and AGO9; 2) this high enrichment of AGOs in meiocytes enables 24-nt siRNAs to target genic loci despite reduced sequence complementarity; 3) AGO4, AGO6, AGO9, and unexpectedly AGO5, load Pol IV- and CLSY3-derived siRNAs in meiocytes; and 4) AGO4/6/9 mediate DNA methylation, whereas AGO5 mediates post-transcriptional gene silencing (PTGS), with these two pathways acting synergistically to regulate protein-coding genes and silence transposons in the germline. No prior papers, according to our knowledge, have reported any of these findings.

To address how these AGOs contribute to meiotic regulation, we performed meiotic phenotyping of *ago5* and *ago4,6,9* triple mutants. Significant meiotic defects were observed in both mutants, demonstrating these pathways are functionally important for meiotic regulation. These results are described in detail in our response to Major Point 7.

With respect to the discrepancy with recent findings in sperm cells, AGO5 has indeed been shown to associate with 21-22 nt TE-derived siRNAs and to mediate DNA methylation in sperm cells (Herridge et al. 2025 NSMB). In contrast, our data demonstrate that AGO5 performs a distinct, stage-specific role in meiocytes, where it predominantly binds 24-nt siRNAs and mediates silencing via a DNA-methylation-independent mechanism (this will be further discussed in Major Point 1 below). Thus, rather than contradicting prior work, our findings uncover a stage-specific functional shift of AGO5 between meiocytes and sperm cells, highlighting previously unrecognized functional diversification of AGO5 during male germline development. We have now compared our findings with the previous report and incorporated this comparison into the revised Discussion (Lines 432-436).

Finally, we agree that genetic analysis would further strengthen the synergistic regulatory role of AGO5 and AGO4,6,9. To address this, we have tried to obtain an *ago4,5,6,9* quadruple mutant. However, due to the genetic linkage between *AGO4* and *AGO5*, we were unable to recover the quadruple mutant despite multiple tries and screening thousands of F2s. Nevertheless, several observations support functional synergy between these pathways. Genes upregulated in *ago5* and *ago4,6,9* meiocytes largely overlap, and *pol-iv* mutation causes substantially more upregulated genes than either mutant alone, besides those that are shared between *ago5*, *ago4,6,9* and *pol-iv* (Fig. 5d). In addition, the genes that are categorized

as only upregulated in *pol-iv* also show mild upregulation in both *ago5* and *ago4,6,9* (Fig. 5e, 5f). Because AGO5- and AGO4,6,9-bound sRNAs are both dependent on Pol IV, these results support the synergistic role of AGO5 and AGO4,6,9 pathways. Nevertheless, we have softened our wording regarding pathway synergy in the revised manuscript (e.g. Line 353-355).

Major concerns:
1. 1. According to the Methods section (page 18), all RIP-seq data were derived from flower buds with only open flowers removed. Notably, AGO4, AGO6, AGO9, and AGO5 are also expressed in later-developed pollen and female gametophytes. More importantly, RIP-seq data of AGO5 and AGO9 in sperm cells reported by Herridge et al. (2025, NSMB) show that AGO5 primarily binds 20–22 nt sRNAs, which contradicts the results here (Fig. 2a). This raises questions about whether the RIP-seq profiles truly represent AGO5 in meiocytes, as the sRNA pool bound by AGO5 might be contaminated by signals from other cell types.

We thank the reviewer for raising this important point. AGO5 RIP-seq data from pollen (Herridge et al. 2025 NSMB) indeed shows that AGO5 primarily binds 20-22nt sRNAs. This is indeed different from our RIP-seq data from floral buds, which shows that AGO5 primarily binds to 24 nt and 21-22 nt sRNAs, with 24 nt being the dominant species. Within floral buds, AGO5 is only expressed in carpels, male meiocytes and sperm cells. As only unopened floral buds (up to Flower Stage 12) were collected for our RIP-seq, sperm-containing mature pollen (produced at Flower Stage 13) was rarely included. Therefore, the comparison of our data and Herridge et al.'s pollen data shows that 24 nt sRNAs indeed bind to AGO5 in meiocytes and carpels. We would like to emphasize that it is not feasible to perform AGO5 RIP-seq on isolated meiocytes due to the limited materials. Moreover, the expression of AGO5 in carpels does not compromise our analysis, because our primary goal is to understand the relationship between AGO5-associated sRNAs and DNA methylation, and it is clear from recent literature that similar DNA methylation is driven by CLSY3-mediated 24-nt sRNAs in anthers and carpels (Zhou et al. 2022 Nat Comm).

To further test how well our AGO5 RIP-seq profiles represent AGO5 in meiocytes*,* we analyzed the percentages of AGO-bound sRNAs that are derived from HyperTE loci. As shown in the table below, 38% of AGO5-bound sRNAs are mapped to HyperTE loci, even higher than AGO4/6/9-bound sRNAs and is comparable to the 40% of meiocyte sRNAs that are mapped to HyperTE loci. Similarly, the percentages of AGO5-bound sRNAs that have potential to map to MetGenes are also comparable to those of meiocyte sRNAs. Consistent with the inclusion of female tissues, 31% of AGO5-bound siRNAs are also mapped to Siren loci. This result demonstrates that our RIP-seq data represents AGOs well in meiocytes.

	**Percentage mapped to MetGene loci (≤ 3 mismatches)**	**Percentage mapped to HyperTE loci**	**Percentage mapped to Siren loci**
**AGO4 RIP-seq**	0.7%	17.8%	21.1%
**AGO5 RIP-seq**	1.3%	37.9%	30.5%
**AGO6 RIP-seq**	0.9%	25.8%	16.9%
**AGO9 RIP-seq**	0.6%	17.1%	9.5%
**Meiocyte sRNA-seq**	2.1%	39.8%	1.0%

2. In P7 and the discussion section, the authors claim twice that "the increased dosage of Pol V pathway components could contribute to the pronounced off-target methylation effects." While Fig. 1c shows that Pol V and AGO4/6/9 are indeed enriched in meiocytes, there is no

evidence demonstrating that overexpression of Pol V or AGO4/6/9 increases off-target methylation effects in somatic cells.

We appreciate the reviewer's careful evaluation of this statement. As the reviewer noted, Pol V and AGO4, 6, 9 are strongly enriched in meiocytes (Fig. 1c). Importantly, we provide functional evidence that the dosage of AGO4, 6, 9 influences off-target DNA methylation at MetGenes. As shown in Fig. 4c, single *ago4, 6* or *9* mutation display only mild reductions in MetGene methylation, but this effect is markedly elevated in double mutants, and finally MetGene methylation is completely abolished when all three proteins are lost in the triple mutant. These results demonstrate that AGO4, 6, 9 act in a semi-redundant manner and show that a high combined dosage of these AGO proteins is required for robust off-target methylation. We have clarified this interpretation and softened our claim in the revised manuscript (Lines 303-306).

We agree with the reviewer that direct evidence showing that overexpression of Pol V and AGO4/6/9 in somatic cells is sufficient to induce off-target methylation is currently lacking. Ideally, this would require coordinated overexpression of the entire Pol V branch of the RdDM pathway, which comprises more than 20 components and is not experimentally tractable. Consistent with this limitation, our model (Fig. 1d) does not propose that overexpression of a single component, such as the largest subunit of Pol V or AGO4/6/9 alone, would be sufficient to trigger off-target methylation in somatic tissues. Rather, we suggest that the concerted upregulation of the entire Pol V branch of the RdDM pathway in meiocytes compensates for reduced affinity due to siRNA-scaffold mismatches and allow off-target methylation (Lines 419-421).

3. In Figure 3a, do Pol IV and CLSY3 affect AGO5 protein abundance?

We thank the reviewer for raising this excellent point. Accordingly, we examined AGO5-Venus signals in *pol iv* and *clsy3* mutants and compared with those in WT background. We did not observe any reduced signal in either mutant. This result has been added to the revised manuscript as Fig. S3a and described in the Results section (Lines 252-255; 264-266). In addition, the reduction of AGO5-bound sRNAs in *pol iv* and *clsy3* mutants shown in Fig. 3a is consistent with our finding that the vast majority of AGO5-bound sRNAs are derived from Pol IV and CLSY3 (and to a less extent, CLSY4) dependent clusters (Fig. 2b,d, S2a,b).

4. The results in Fig. 4 demonstrate functional redundancy but not a "dosage-dependent role" of AGO4, AGO6, and AGO9.

We thank the reviewer for this comment. As discussed in our response to Point 2, the progressive enhancement of MetGene methylation defects from single to double and ultimately triple *ago4,6,9* mutants (Fig. 4c) indicates not only functional redundancy but also a requirement for a high combined dosage of AGO4, AGO6, and AGO9 for robust off-target methylation. We have softened our claim and clarified this interpretation in the revised manuscript (Lines 303-306).

5. In Fig. 6d, the upregulated TEs in ago4,6,9 do not overlap well with those in pol-iv, whereas the DEGs (Fig. 5a–d) show significant overlap between these two mutants. How can this result be explained? Additionally, the methylation status of those upregulated TEs (Fig. 6g) does not show significant loss.

We thank the reviewer for raising this important point. Indeed, while about 60% of upregulated genes in *ago4,6,9* overlap with those in *pol-iv*, but for TEs, our analysis shows about only 20% of upregulated TEs overlap. We believe this is because TE upregulation by the loss of RdDM

is relatively mild but a strict cutoff was applied (*q*-value < 0.01 and Fold change >=4). When we apply less stringent cutoffs (see the Venn diagrams below), the overlap between upregulated TEs in *ago4,6,9* and *pol-iv* increases substantially, indicating that many TEs are transcriptionally affected in both mutants but fall below the strict threshold in one condition. We have clarified in the revised manuscript that a substantial fraction of TEs categorized as '*pol-iv* only' or '*ago4,6,9* only' in fact show increased transcription in both backgrounds (Lines 389-392 and 398-403 and Fig. 6f,g). To further substantiate this point experimentally, we examined At2TE23670 (the Group2 example in Fig. 6g), a TE classified as '*pol-iv* only', using reporter lines. This analysis revealed clear transcriptional activation in both *ago4,6,9* and *pol-iv* meiocytes. These data are now included as a new Fig. S7.

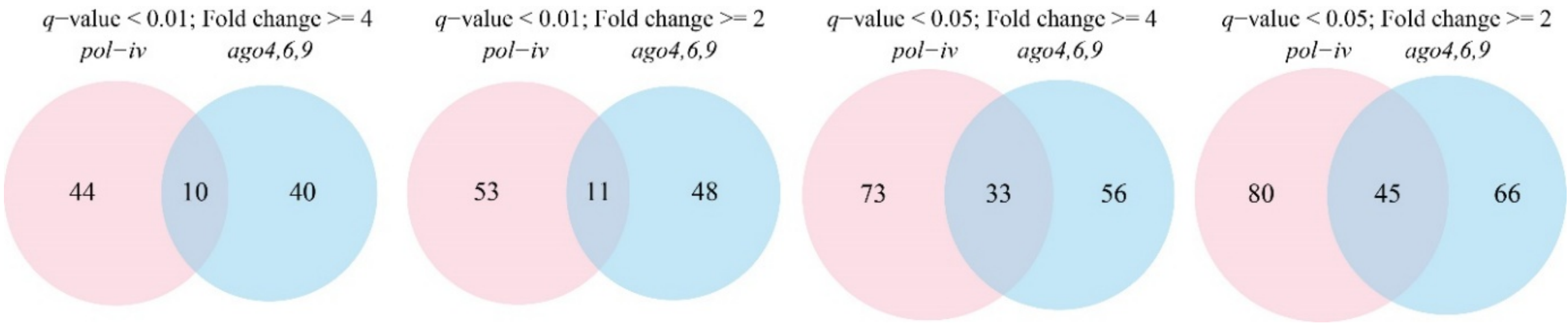

The upregulated TEs indeed show significant loss of DNA methylation in *ago4,6,9* and *pol-iv* mutants (Fig. 6g). These are typically LTR-retrotransposons, whose derepression is known to result from loss of methylation at LTR regions (Galindo-González et al. Gene 2017; Long et al. Science 2021). We apologize for not making this clear in the previous figure. In the revised figure, we have highlighted the differentially methylated regions using shading to make this effect explicit (revised Fig. 6g).

6. In the ago5 mutant, what might explain the higher number of downregulated genes compared to upregulated genes? Furthermore, the only upregulated TE in the ago5 mutant is AT5TE50260, which belongs to LTR/Copia. Does this TE belong to the HyperTE loci?

On large, the *ago5* mutation alone has a relatively mild effect on gene expression. When we use a stringent cutoff (*q*-value < 0.01), we observe more downregulated genes than upregulated genes (Fig. 5a; also shown in the left panel of Figure A below). However, when we apply less stringent cutoffs, such as *p*-value < 0.01 or < 0.05, the numbers of down- and up-regulated genes become similar (middle panel in Figure A), or shift toward a higher number of up-regulated genes than down-regulated genes (right panel in Figure A). Therefore, the relative numbers of down- versus up-regulated genes are sensitive to the chosen statistical cutoff.

To more robustly assess the directionality of misregulation, we next examined effect size by plotting the absolute values of log2 fold changes (*ago5*/WT). As shown in Figure B below, upregulated genes display significantly larger fold changes than downregulated genes across all *p*- and *q*-value cutoffs tested. Therefore, on large, the *ago5* mutation leads to stronger gene upregulation than downregulation.

AT5TE50260 is not a HyperTE locus, consistent with the role of AGO5 in PTGS instead of TGS.

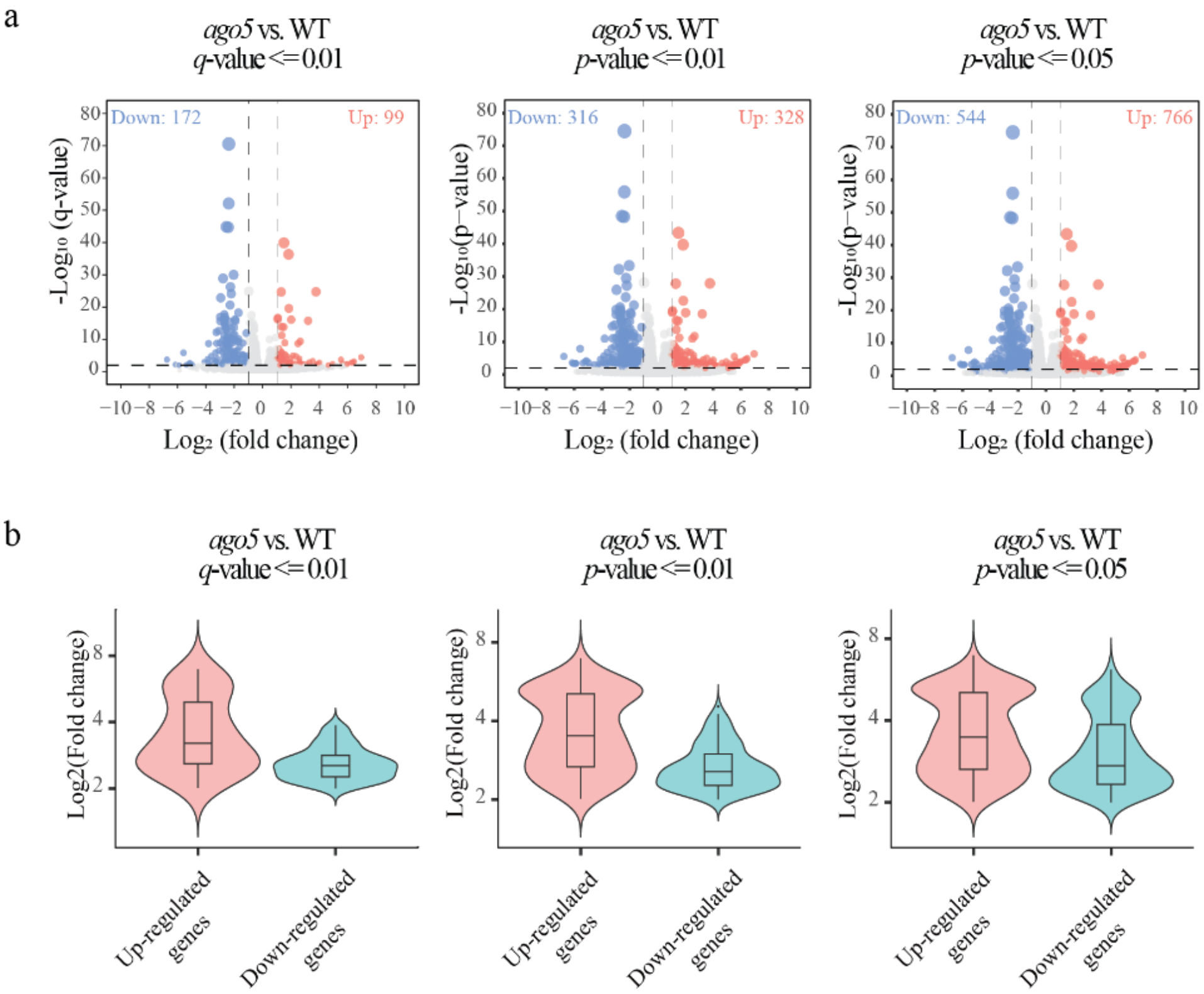

*7. Most importantly, do ago5, *ago4/6/9*, and pol IV mutants exhibit any defects in meiosis and subsequent male germ cell development? The corresponding author's lab has previously reported meiotic abnormalities in RdDM mutants in their Nature Genetics (2018) paper.*

We thank the reviewer for this excellent suggestion. We have examined the meiotic phenotypes of *ago5*, *ago4,6,9* triple and *pol-iv* mutants as requested, and alongside, we have also included WT and *drm1 drm2* mutants as controls. The results are shown in Table S5 and described in the revised manuscript:

Line 329-336: "Previously, we showed that this *de novo* methylation pathway promotes male meiosis, as the *drm1 drm2* double mutant produces an increased proportion of triads at the end of meiosis compared to WT (7% in *drm1 drm2* versus 0% in WT)[44]. Consistently, we found both *ago4,6,9* triple mutant and *pol-iv* mutant exhibited elevated frequencies of triads relative to WT (Extended Data Fig. 5c and Supplementary Table 5). The penetrance of this phenotype was modestly lower than previous reported for *drm1 drm2* mutant (4% versus 7%), likely reflecting differences in growth conditions, which are known to influence RdDM activities[53-56]."

Lines 349-351: "Supporting a role for AGO5 in regulating meiocyte gene expression, the *ago5* mutant also displayed a modest but significant increase in triad formation (Extended Data Fig. 5c and Supplementary Table 5)."

Reviewer #2 (Remarks to the Author):

In this manuscript, Long et al investigate the functions and biogenesis of siRNAs and argonautes in Arabidopsis male meiocytes (cells undergoing meiosis). They first identify the four most strongly expressed Ago genes in meiocytes and then carry out RIP-seq for each of the four to characterize their associated siRNAs. They focus on one, Ago5, that behaves differently than the others, and they carry out RIP-seq on Ago5 in multiple different mutant backgrounds including double mutants. This is a major accomplishment. They also carry out RNA-seq in the in an Ago triple mutant and in the single Ago5 mutant to investigate effects on both gene and TE expression. The major conclusions of the study focus on siRNAs that were previously reported to be transcribed by pol IV in the surrounding tapetal cells and transferred into meiocytes where they direct gene silencing even with up to three mismatches between siRNAs and targets. Long et al call the target genes "MetGenes". They propose an interesting model for why silencing with mismatches could be tolerated in meiocytes but not in other cells: spatial separation of siRNA synthesis from siRNA targeting prevents amplification of the silencing signal, which would otherwise cause an off-target silencing problem or a heritable silencing problem for mismatched siRNA targeting. The work is extensive and detailed and appears to have been done rigorously.

We thank the reviewer for the positive evaluation of the significance, novelty and rigor of our study.

I have one major concern regarding lack of direct evidence to connect specific siRNAs and Agos with differential gene expression in mutants. While previous work has already reported that pol IV-derived siRNAs can target MetGenes, and Long et al demonstrate that Ago5 binds pol IV-derived siRNAs, they do not determine whether Ago5 siRNAs have the potential to target MetGenes. An analysis should be included that shows the proportion of siRNAs from each Ago that have the potential to target MetGenes (allowing up to three mismatches), and the distributions of targeting-siRNAs per gene. Similarly, if differentially expressed (DE) genes that Long et al have identified in this manuscript are directly regulated by ago4,6,9 or ago5, the siRNAs from these argonautes should match the DE genes, independently of whether they qualify as MetGenes. This should be tested by plotting the distribution of siRNA abundance for DE genes from each ago RIP-seq dataset. This should be done separately for downregulated, upregulated, and non-DE genes. Paralell analyses should be done for TEs as well. This will allow a better way to evaluate the potential significance of Ago5 in terms of gene regulation, as well as the other three Agos.

We thank the reviewer for these constructive and insightful suggestions. First, we performed analyses on AGO4, 5, 6, 9-bound siRNAs as suggested (added to the revised manuscript as a new Extended Data Fig. 3b). We found that siRNAs associated with all four AGOs, including AGO5, are significantly enriched at MetGene loci (with up to three mismatches), but not at a random set of loci or all genes. Importantly, the distribution of AGO-bound siRNA abundance per gene for MetGenes shows that targeting is broadly distributed across MetGenes rather than being driven by a small number of outlier genes (Extended Data Fig. 3b). This result is consistent with our observation that AGO5, like AGO4, 6 and 9, preferentially binds Pol-IV- and CLSY3-dependent siRNAs, supporting the conclusion that AGO5-bound siRNAs have the potential to target MetGenes.

As suggested by the reviewer, we further calculated the proportion of 24-nt siRNAs from each AGO RIP-seq dataset that can potentially target MetGenes (with up to 3 mismatches). The results are displayed in the table below. About 1.3% of AGO5-bound siRNAs have the potential to target MetGenes, compared to 0.7%, 0.9% and 0.6% for AGO4-, AGO6- and AGO9-bound siRNAs, respectively. For comparison, in male meiocytes, ~2.1% of 24-nt

siRNAs have the potential to map to MetGenes (Long et al. 2021 Science). As AGO RIP-seq was performed using young floral buds containing both male and female tissues, and carpel-derived siRNAs do not map to MetGenes (indeed, as shown in the table below, ~10-30% AGO4,5,6,9-bound siRNAs are perfectly mapped to siren loci), the observed proportions demonstrate the quality of our RIP-seq data and that AGO5-bound siRNAs have similar potential to target MetGenes, compared to AGO4, 6 and 9.

	**Percentage mapped to MetGene loci (≤ 3 mismatches)**	**Percentage mapped to HyperTE loci**	**Percentage mapped to Siren loci**
**AGO4 RIP-seq**	0.7%	17.8%	21.1%
**AGO5 RIP-seq**	1.3%	37.9%	30.5%
**AGO6 RIP-seq**	0.9%	25.8%	16.9%
**AGO9 RIP-seq**	0.6%	17.1%	9.5%
**Meiocyte sRNA-seq**	2.1%	39.8%	1.0%

For the second point, first we agree that not all differentially expressed (DE) genes are directly regulated by *ago4,6,9* or *ago5* (Lines 325-326). These DE genes were identified by RNA-seq analysis of WT and *ago* mutants, and therefore include genes that are directly regulated by AGO, as well as their downstream genes whose expression are indirectly altered as a consequence of regulatory network effects. As requested, we therefore plotted the distribution of AGO-bound siRNA abundance per gene separately for upregulated DE genes, downregulated DE genes and non-DE genes for each AGO RIP-seq dataset (Figure A-D below). We observed significant enrichment at both upregulated and downregulated DE genes, in comparison to non-DE genes. This pattern was observed for AGO5, as well as for AGO4, 6 and 9, supporting a direct contribution of these AGOs to gene regulation.

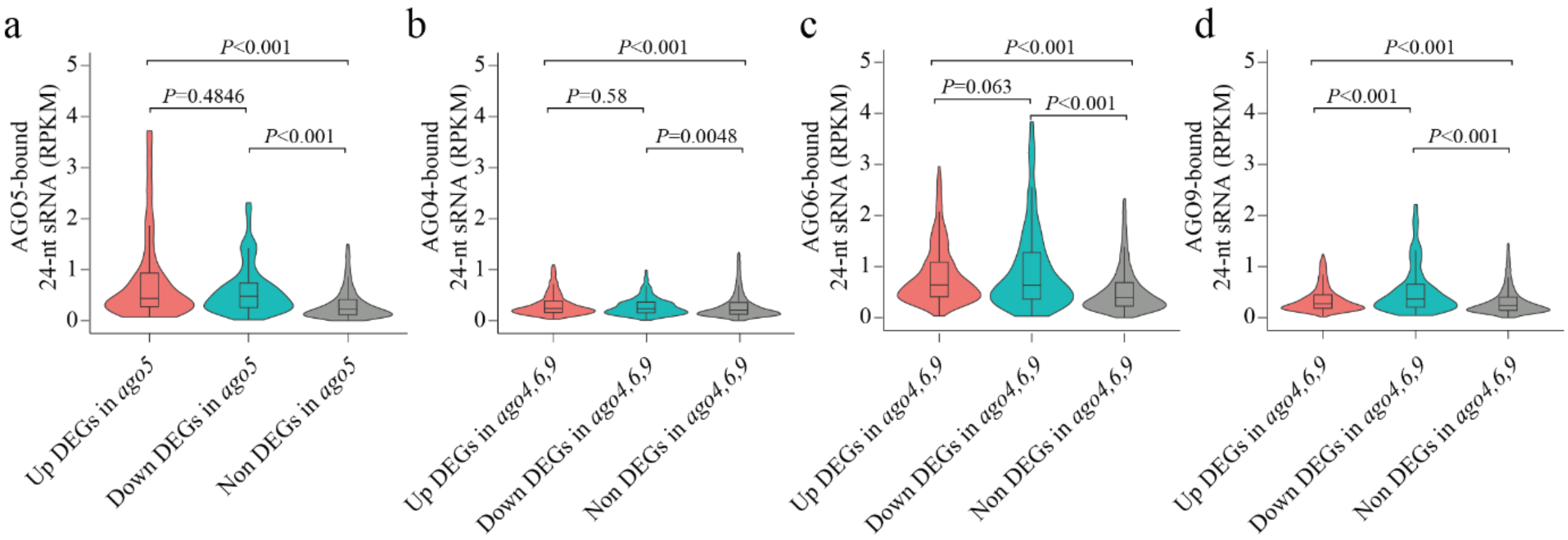

We performed analogous analyses for TEs (Figure A-C below). Unlike genes, AGO4, 6, 9-bound siRNAs were significantly enriched at TEs that are upregulated in *ago4,6,9* triple mutant (compared to WT), in comparison to TEs that are downregulated, or TEs that are not differentially expressed (non-DETE). This shows that these TEs are directly suppressed by AGO4, 6 and 9, consistent with our knowledge of TE regulation by siRNAs. We could not perform this analysis on AGO5, as only 1 TE is misregulated in *ago5* mutant.

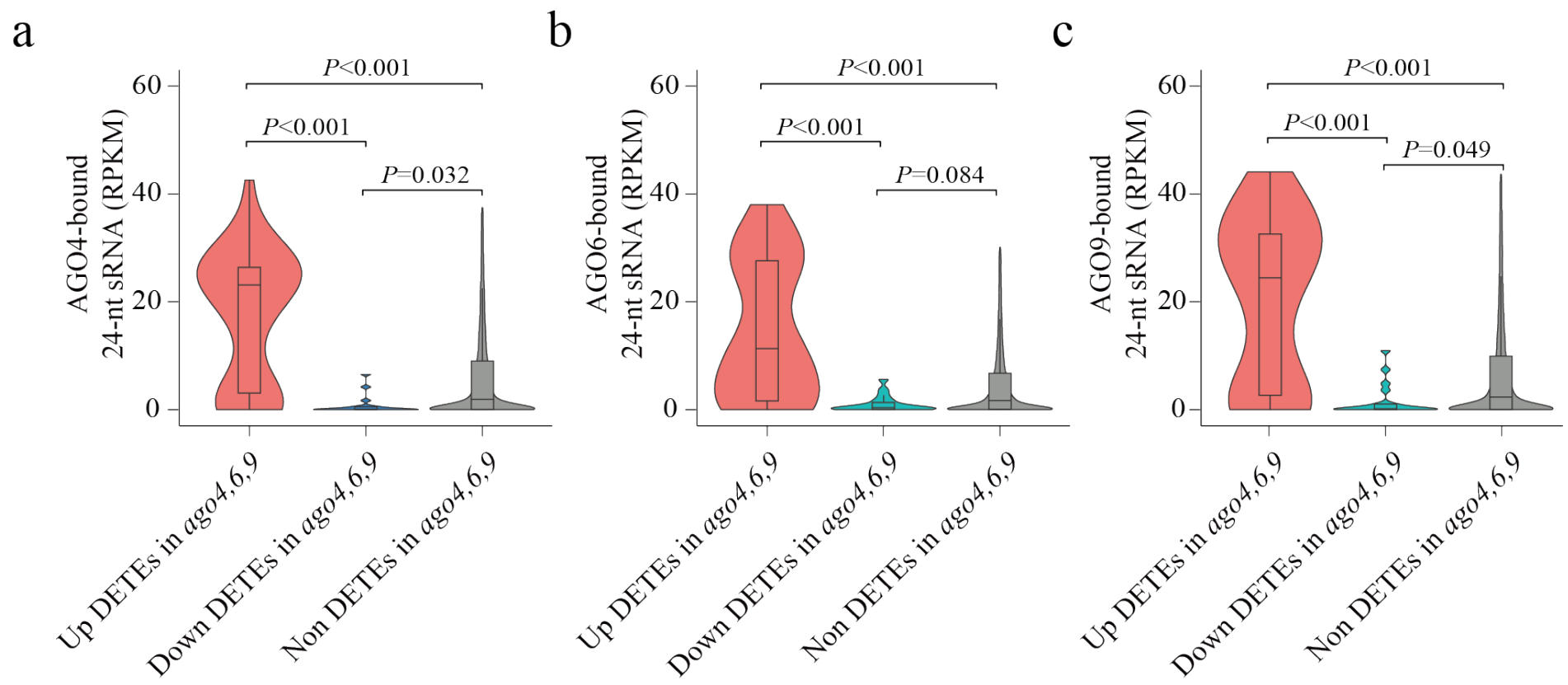

A moderate concern is that in many places the text seems overly qualitative. In places where I would expect numbers there are just descriptive terms. Could words like "preferentially", "a subset", "a fraction", "largely", "most", and "slight" be supplemented with numbers that define them? Or is the source data not accurately quantifiable?

We thank the reviewer for this helpful suggestion. We have revised the text to replace some of the qualitative descriptors with quantitative values where doing so clearly improved precision without sacrificing readability (e.g. Lines 210-213, 390-391, 398, 403-404, 405-406). In other cases, we have retained qualitative terms, as replacing all such instances with numerical values would substantially lengthen the sentences and reduce readability without adding meaningful information.

Of lesser concern;

The title is not particularly informative. Don't Multiple Argonaute complexes orchestrate epigenetic silencing in any tissue?

Our manuscript reports several new, distinct findings: we show that (1) male reproductive siRNAs associate with multiple Argonaute proteins, including AGO4, AGO5, AGO6, and AGO9; (2) these AGOs are highly enriched in meiocytes, and this enrichment enables siRNAs to target genic loci with reduced sequence homology; (3) AGO4, AGO6, AGO9, and unexpectedly AGO5, load Pol IV-derived siRNAs and target genic loci with mismatches; and (4) AGO4/6/9 mediate DNA methylation, whereas AGO5 mediates post-transcriptional gene silencing, with these two pathways acting synergistically to regulate protein-coding genes and silence transposons in the germline.

Given the breadth of these findings and their mechanistic diversity, we believe that a highly specific title would not adequately capture the full scope of the study. We therefore chose the title *"Multiple Argonaute complexes orchestrate epigenetic silencing in the Arabidopsis germline"*, which we feel best reflects the integrative nature of the work while remaining accessible to a broad readership.

A bit more background on MetGenes is needed to understand this paper. How many have been identified? How many are differentially expressed in siRNA mutants and methylation mutants in meiocytes? What parts of the genes are differentially methylated between tapetum and meiocytes? Coding DNA? Introns? Promoters? Is the differential methylation in all three major contexts (CHH, CHG, and CG)?

We thank the reviewer for the great suggestion. MetGenes refer to the target loci of tapetal 24-nt siRNAs via reduced sequence complementarity (with up to 3 mismatches). These loci are hypermethylated in the meiocytes in all sequence contexts (CG, CHG and CHH), compared to tapetal or somatic cells, and mostly overlap gene coding regions (> 63% of MetGenes overlap gene bodies), and therefore we abbreviated these loci as MetGenes. There are 469 MetGenes in the Arabidopsis genome, and out of these 11 have been demonstrated to cause differential gene expression in RdDM mutant meiocytes. All of these information are available in Refs 43 and 44, but we agree that they are also helpful for this manuscript and have therefore revised the introduction and incorporated this information.

Is the published RNA-seq meiocyte data from the same stage as meiocytes prepared for this study? What stage is that?

The published RNA-seq meiocyte data (Walker et al. 2018 Nat Genet; Long et al. 2021 Science) are from the same stage and isolated using the same method as used in this study. It is meiotic prophase I. We have added this information to the Methods.

Specify whether Venus Ago fusion genes were encoded with full-length Ago genomic DNA including introns or just CDS only.

Full-length Ago genomic DNA (including introns) were used. We have specified this in the Methods accordingly.

Figure 1: Error bar sizes? Define RFP-TAU5. Panel D is a little confusing becuase it makes it look like the siRNAs that target MetGenes are secondary siRNAs derived from HyperTEs in the meiocyte rather than siRNAs transferred from the tapetum.

We thank the reviewer for pointing these out. Accordingly, we have added the scale bar information to the legend of Fig. 1, and revised Fig. 1D to clarify that the siRNAs targeting MetGenes are those transferred from the tapetum. *RFP-TAU5* refers to *RFP-TUBULIN ALPHA-5*, which labels the microtubule cytoskeleton (from Ref 66). This information was previously described in the Methods section (Lines 472-473), but we have also added this to the figure legend for clarity.

Figure 2: The categorization schemes and labels in panels C and D are hard to understand. Maybe more detail in the legend would fix this, but it may require rethinking the figure. Similar problem in Figure 3D.

We thank the reviewer for bringing this to our attention. We agree and have revised Figs. 2C, 2D, 3D and Supplementary Fig. 2b, as well as the corresponding figure legends accordingly.

It is also hard to understand the Y axis scale in Figure 3B. What are the values normalized by? The legend indicates the values are "relative to the total number derived from pol-iv-dependent clusters". But if this is true, the total sum of the values for pol-iv-dependent clusters should be constant across genotypes, including in the pol iv mutant itself. What the plots in fact show is near-zero values for pol-iv-dependent clusters for the pol iv mutant, which suggests something else is being used as the normalizing factor.

We thank the reviewer for pointing this out. The numbering in the figure and the legend was incorrect and has now been corrected. The Y axis in the previous Figure 3B (current Figure 3C) refers to "the levels of AGO5-bound sRNAs at indicated *clsy* mutant dependent clusters". The values that are "relative to the total number derived from *pol-iv*-dependent clusters*"* are

shown in the previous Figure 3d (current Figure 3b), and indeed the total sum of the values are all 100%.

In Figure 4, the number of loci included in each distribution should be indicated. Also it's not clear what the top colored segments in panel B are showing.'

We thank the reviewer for this suggestion. In the figure legend, we have now indicated the number of loci included in each distribution and clarified that the top colored segments in panel B represent HyperTE (blue color) or MetGene (pink color) loci.

While 50 TEs are upregulated in ago4,6,9 vs. WT, 21 TEs are also downregulated. Since siRNAs are predicted to suppress gene expression, this suggests that 2/5ths of the differential expression in this triple mutant is indirect. It would be worth mentioning this caveat in the interpretation. Also related to interpretation, even if one generously includes all 104 upregulated TEs in the number, does that support the statement "AGO4/6/9 and Pol IV are key regulators of TE silencing in meiocytes"? If the vast majority of TEs are not activated in the mutants, is it fair to call the Agos "key regulators"? Could one conclude the opposite, that they are largely; dispensable regulators in meiocytes?

We thank the reviewer for bringing this to our attention. We re-examined the 21 TEs that were initially classified as downregulated in *ago4,6,9* triple mutant. We found that 7 out of these TEs showed high RPKMs in both *ago4,6,9* and WT samples, which were driven by mapping artefacts over poly(A/T) sequences in these TEs. We therefore refiltered the RNA-seq data to exclude TEs whose coverage was dominated by low-MAPQ multi-mappers and secondary alignments over poly(A/T) tracts. The updated differential expression analyses are displayed in revised Fig. 6a-c. Importantly, the upregulated TEs were not affected by this filtering, whereas the number of downregulated TEs in ago*4,6,9* was reduced from 21 to 14. Added together, there are 104 upregulated versus 17 downregulated TEs. In addition, we note that our analysis used a relatively stringent threshold to define TE derepression ($q < 0.01$ and fold change > 4). Using a more commonly applied cutoff ($p < 0.05$ and fold change > 2) increases the number of upregulated TEs from 104 (in *ago4,6,9* and *pol-iv* combined) to 236.

We agree that not all transcriptional changes are expected to reflect direct siRNA-mediated repression. However, the strong enrichment of AGO4/6/9-bound siRNAs at upregulated TEs (see response to Major Point 1 on Page 8), together with the observed methylation loss at these loci (Fig. 6g), supports direct regulation of this subset. To further validate the derepression detected by RNA-seq, we utilized a reporter line for one of the TEs that is shown to be upregulated in *pol-iv* (but not detected as significantly upregulated in *ago4,6,9* mutant) by RNA-seq, AT2TE23670 (shown as an example of Group 2 TEs in Fig. 6g). This TE shows significant loss of DNA methylation at its LTRs (Fig. 6g). We observed clear activation of AT2TE23670 in germlines of both *pol-iv* and *ago4,6,9* mutants (added as a new Fig. S7), showing that this TE is directly regulated by AGO4/6/9 and Pol IV through DNA methylation.

Importantly, in Arabidopsis somatic tissues, loss of RdDM leads to widespread reduced CHH methylation in the genome but transcriptional derepression is restricted to very few TEs. In contrast, our results show that in meiocytes, loss of AGO4/6/9 and Pol IV results in more pronounced TE activation, indicating that RdDM plays a more prominent functional role in TE repression in the germline than in somatic tissues. We further note that Arabidopsis has relatively low TE content (~15% of the genome) compared to many crop species (e.g. maize, ~85%). Therefore, even moderate levels of TE derepression in Arabidopsis may underestimate the potential impact of disrupting analogous pathways in TE-rich genomes. We have revised the Discussion to clarify this point.

Spatial separation of siRNAs from their Ago proteins would make them susceptible to degradation by nucleases. Do Long et al have any ideas on how the siRNAs avoid degradation in tapetal cells before transfer to the meiocytes for loading onto Agos?

At present, we do not know how these siRNAs are transported from tapetal cells to meiocytes, nor in what molecular form they are transported. One possibility is that they are transported as double-stranded RNA duplexes, which would confer increased resistance to nuclease-mediated degradation, potentially in association with RNA-binding proteins or vesicular transport. How this protection is achieved remains an intriguing open question beyond the scope of this study.

Does the Ago triple mutant, the Ago5 single mutant, or the pol IV single mutant have a male fertility defect? Either way, this should be mentioned in the text.

We thank the reviewer for this excellent question. We have examined the meiotic phenotypes of *ago5*, *ago4,6,9* triple and *pol-iv* mutants as suggested, and alongside, we have also included WT and *drm1 drm2* mutants as controls. The results are shown in Table S5 and described in the revised manuscript:

Line 329-336: “Previously, we showed that this *de novo* methylation pathway promotes male meiosis, as the *drm1 drm2* double mutant produces an increased proportion of triads at the end of meiosis compared to WT (7% in *drm1 drm2* versus 0% in WT)[44]. Consistently, we found both *ago4,6,9* triple mutant and *pol-iv* mutant exhibited elevated frequencies of triads relative to WT (Extended Data Fig. 5c and Supplementary Table 5). The penetrance of this phenotype was modestly lower than previous reported for *drm1 drm2* mutant (4% versus 7%), likely reflecting differences in growth conditions, which are known to influence RdDM activities[53-56].”

Lines 349-351: “Supporting a role for AGO5 in regulating meiocyte gene expression, the *ago5* mutant also displayed a modest but significant increase in triad formation (Extended Data Fig. 5c and Supplementary Table 5).”

We thank the reviewers for their constructive comments. We have performed additional experiments and analyses to address these comments, which we believe have significantly improved the manuscript. Below we reproduce each of the reviewers' comments in italics, followed by our response.

Reviewer #1 (Remarks to the Author):
In this revision, the authors have included additional experimental results, particularly refined AGO5 RIP-seq comparisons, new evidence supporting Pol IV–dependent DNA methylation, Venus-AGO5 imaging in pol IV and clsy3 backgrounds, reporter-based validation, and improved data analysis. Moreover, they have added explanations to clarify several previously confusing observations. These additions strengthen the study, and my previous concerns have been largely addressed. I therefore recommend the manuscript for publication, with the following points to be addressed:

Considering the very weak meiosis phenotype in the corresponding mutants, please provide a brief GO analysis (indicating which specific meiotic processes the deregulated genes are involved in) for the genes identified as deregulated (e.g., MetGene) in these mutants. The biological significance of these deregulated genes should also be discussed.

We've added the GO analysis into the revised manuscript as Extended Data Fig. 5b. In general, photosynthesis-related GO terms are enriched among the upregulated genes in all *ago5*, *ago4,6,9* and *pol-iv* mutants; no meiosis-related GO terms are consistently enriched among the differentially expressed genes, which is consistent with our previous report on *drm1drm2* mutant vs WT meiocyte RNA-seq data comparison (Walker et al. 2018 Nat Genet).

However, the meiosis phenotype in RdDM mutants is primarily caused by the mis-splicing of a key meiotic gene, *MPS1/PRD2* (Walker et al. 2018 Nat Genet). Loss of methylation in *MPS1*'s last intron causes elevated intron retention, which introduces a pre-mature stop codon and reduces the functional *MPS1* transcript levels (Walker et al. 2018 Nat Genet). Consistent with this, we detected elevated intron retention in *ago4,6,9* and *pol-iv* mutant meiocyte RNA-seq datasets, compared to WT. This result was further validated by RT-PCR (added as Extended Data Fig. 6a), and nicely explains the *ago4,6,9* and *pol-iv* meiotic phenotypes. Although *ago5* does not detectably affect DNA methylation, we also found elevated intron retention in *ago5* mutant meiocytes by RNA-seq and RT-PCR (Extended Data Fig. 6a), suggesting a partial convergence with meiotic regulation with AGO4,6,9 and Pol IV. We have added the new data and analysis to the revised results section (Lines 338).

Please ensure that all figure legends involving statistical plots (e.g., violin plots, box plots) consistently report statistical methods, sample sizes, and p-value calculations throughout the manuscript. For example, the figure legend for Extended Data Fig. 3b should include the statistical test used, a description of how p-values were calculated, and a clear indication of what the box plot elements represent (e.g., median, quartiles).

We thank the reviewer for this helpful comment. We have revised the figure legends throughout the manuscript to consistently report the statistical methods, sample sizes,

and *P* value calculations for all relevant statistical plots. For example, the legend of Extended Data Fig. 3b now specifies the statistical test used, how p-values were calculated, and the definitions of the box plot elements. Similar information has been added or clarified in Fig. 3c, Fig. 4, Fig. 5f, Fig. 6f, and Extended Data Figs. 3–6.

Briefly clarify in the Results or Discussion section why different statistical thresholds lead to substantial differences in transposable element (TE) overlap, to help guide interpretation and avoid potential confusion.

Accordingly, we have included the Venn diagrams in the revised manuscript (Extended Data Fig. 7d), showing progressively increased overlap between upregulated TEs in *ago4,6,9* and *pol-iv* when less stringent cutoffs are applied. As in the current results session, we already discuss that a substantial fraction of TEs categorized as '*pol-iv* only' or '*ago4,6,9* only' in fact show increased transcription in both backgrounds. To avoid repetition and interruption of the flow of the Results section, we incorporated the requested discussion regarding the influence of statistical thresholds into the figure legend.

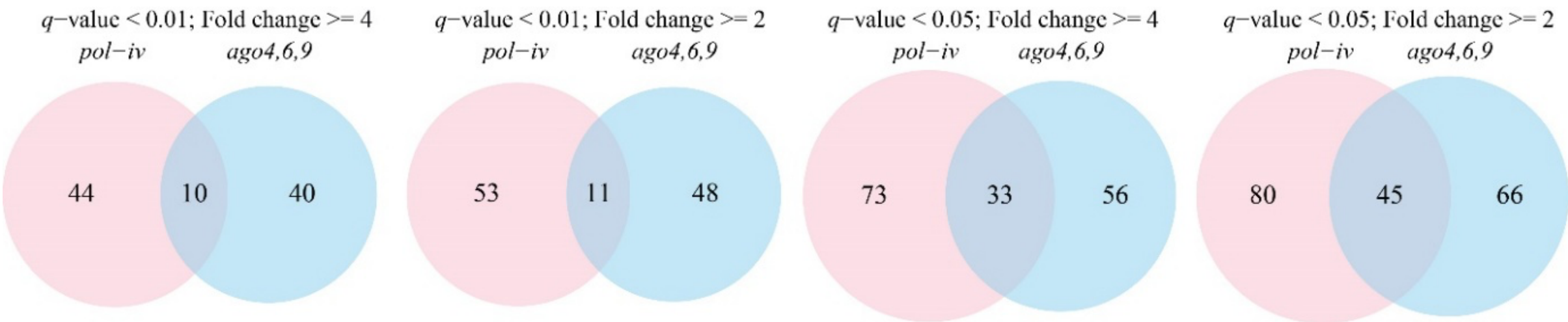

Reviewer #2 (Remarks to the Author):
Long et al have mostly addressed my previous concerns in their rebuttal. I am surprised though that some of the data they put into the rebuttal they did not also put into or mention in the revised manuscript. In particular, ago mutant DE genes, unlike DE TEs, have similar numbers of Ago-bound siRNAs regardless of whether they are up or down regulated in mutants, and there are relatively small differences in numbers of Ago-bound siRNAs between DE genes and non-DE genes. It seems important that those numbers be included for the reader to consider. Not necessary but helpful would be some discussion too. Why would so many genes be targeted by Ago-bound siRNAs but down-regulated or not affected in ago mutants? It would also strengthen the manuscript to explicitly describe the amount of overlap between MetGenes and each group of DEGs, both up and downregulated ones. The manuscript clearly shows enrichment in overlaps between the different mutant DEGs, but leaves out the enrichment between DEGs and MetGenes. This, along with the above results, would help clarify how relevant the mutant DE analyses are to understanding the MetGene siRNA pathway.

We thank the reviewer for the helpful comments. We have included the AGO-bound siRNA analyses for both differentially expressed (DE) genes and TEs in the revised manuscript (new Extended Data Figs. 5c and 7a) and discussed the results in the Results session (Lines 321-337). In brief, the relatively small differences between non-DEGs and DEGs are likely because only a small proportion of DEGs are directly affected by DNA methylation, whereas most of them are indirectly affected. This result is consistent with our previous report of RdDM's effect on meiocyte gene expression (Walker et al. 2018 Nat Genet). In addition, both upregulated and downregulated DEGs are associated with significantly higher levels of AGO-bound sRNAs in comparison to non-DEGs. This observation is consistent with previous studies showing that DNA methylation can either activate or repress gene transcription depending on the genomic context (Williams et al. 2015 PloS Genet; Xiao et al. 2019 JIPB).

As requested, we have examined the overlaps between DEGs and MetGenes, and included these analyses in the revised manuscript (new Extended Data Figs. 5d). These results have also been added to the revised Results section as follows:

Lines 323: "Consistent with the RdDM pathway's prevalent role in transcriptional repression[52], the majority (approximately 75%) of DEGs were up-regulated in *ago4,6,9* and *pol-iv* mutants (Fig. 5b,c). Moreover, up-regulated DEGs in *ago4,6,9* and *pol-iv* meiocytes significantly overlapped with MetGenes, whereas *ago5* DEGs and down-regulated DEGs in *ago4,6,9* or *pol-iv* meiocytes did not show significant overlap (Extended Data Fig. 5d). Nevertheless, the overlap between up-regulated DEGs and MetGenes remained relatively small (~2%; Extended Data Fig. 5d), suggesting that most transcriptional changes are likely indirect consequences rather than direct effects of DNA methylation loss."

Regarding this statement: "genes located near MetGenes loci (<1.2 kb) exhibited a clear trend of transcriptional up- regulation in pol-iv and ago4,6,9 meiocytes, but not

in ago5 meiocytes (Fig. 5g)": Are MetGenes and MetGene loci the same thing? Since there are only 99 Ago5-upregulated DEGs, I would not expect to see an ago5 mutant effect on the entire MetGene set in this kind of metagene analysis. The signal of those 99 genes would be too subtle relative to the ~1000 genes upregulated in the ago triple mutant. Related to this, the significance of Fig. 5g is hard to evaluate. Is it supposed to demonstrate that Ago5 does not impact expression of flanking genes, but Ago4,6,9 do? If so, the analysis should be repeated with just the 99 Ago5-upregulated DEGs instead of all MetGenes. The authors should also consider the possibility that a lot of MetGenes are poorly annotated and their TSSs and polyadenylation sites are incorrect, and they should consider the possibility that some upregulated MetGenes or DEGs are actually misannotated HyperTEs (TEs that are targeted by the same siRNA pathway in meiocytes).

We apologize for the confusion and thank the reviewer for bringing up this point. MetGenes and MetGene loci are the same thing, as MetGenes refer to loci that are targeted by HyperTE-derived 24 nt siRNAs with imperfect sequence similarity (Lines 129-130). "Many of these target loci overlap genes and are hypermethylated in the meiocyte, hence we named them MetGenes (469 loci in total)" (Lines 130-131). To avoid confusion, we have corrected the reference to 'MetGene loci' in the manuscript and consistently used 'MetGenes' to refer to these loci. In essence, MetGenes are not genes, they are methylated loci which largely overlap genes; similarly, HyperTEs are not TEs, they are the source loci of tapetal 24-nt siRNAs, which are hypermethylated in the tapetum and largely overlap TEs (Lines 124-127).

In Figure 5g, we plotted all genes that overlap MetGenes at indicated distances, and the number of genes being plotted at each distance is the same for all three panels. For example, at distance 0 bp, 219 genes that overlap with MetGenes were plotted for all three panels, and then we looked that the differential expression of these 219 genes in *ago5*/WT (top), *ago4,6,9*/WT (middle), and *pol-iv*/WT (bottom). The results show that the genes within about 1.2 kb of MetGenes exhibit upregulation in *pol-iv* and *ago4,6,9*, but not in *ago5*, suggesting the *ago5* mutation has a relatively minor role in MetGene-mediated regulation of gene expression. This result is consistent with our finding that AGO5, unlike AGO4,6,9 and Pol IV, does not contribute to MetGene methylation in meiocytes. It is also consistent with the requested analysis from the previous point, which shows that differentially expressed genes in *ago5* mutant meiocytes do not significantly overlap with MetGenes, unlike genes that are upregulated in *ago4,6,9* and *pol-iv* mutant meiocytes (the new Extended Data Figure 5d).

We agree that incomplete gene and TE annotations may contribute to ambiguity at individual loci. However, as mentioned above, MetGenes and HyperTEs are not defined by existing gene or TE annotations, but rather by their epigenetic and small RNA features. Therefore, potential annotation inaccuracies are unlikely to alter the overall classification of these loci or the genome-wide trends observed in Fig. 5g. Furthermore, because the same set of genes was analyzed across all three genotypes, the resulting profiles remain directly comparable between *ago5*, *ago4,6,9*, and *pol-iv* mutants.

Fig. 3a: Tracks 1 and 3 (WT R1 and clsy1,2 R1) look oddly similar. Is this an error in data analysis, or are these samples indeed so similar?

There are indeed similar, but not the same. The table below lists the 5' terminal nucleotide of AGO5-bound sRNAs and their percentages.

	**G**	**A**	**U**	**C**
AGO5_RIP_WT R1	4.10342	11.293	7.8382	76.7654
AGO5_RIP_WT R2	8.46349	13.2739	9.52297	68.7396
AGO5_RIP_*clsy1,2* R1	16.7839	13.9755	15.4552	53.7853
AGO5_RIP_*clsy1,2* R2	9.97256	26.7198	9.59401	53.7136

About this statement "the ago5 mutant also displayed a modest but significant increase in triad formation"--is it so modest that it cannot be quantified like ago4,6,9 triple mutant and pol-iv mutants? Both of those are reported in the text to have 4% triad formation.

These data are included in Table S5, which shows that the *ago5* mutant exhibits 2% triad formation.
